# *Txnrd2* loss in skeletal muscle causes muscle atrophy and drives leanness and obesity resistance

**DOI:** 10.1016/j.redox.2026.104165

**Published:** 2026-04-08

**Authors:** Claudia Kiermayer, Rebecca Erdelen, Sonja C. Schriever, Ramona Böhm, Cornelia Prehn, Anna Artati, Maximilian Kleinert, Manuel Miller, Kenneth A. Dyar, Roland M. Schmid, Paul T. Pfluger, Jerzy Adamski, Markus Brielmeier

**Affiliations:** a Core Facility Laboratory Animal Services (CF-LAS), Helmholtz Munich, German Research Center for Environmental Health, Neuherberg, Germany; b Research Unit Neurobiology of Diabetes, Institute for Diabetes and Obesity, Helmholtz Munich, German Research Center for Environmental Health, Neuherberg, Germany; c German Center for Diabetes Research (DZD), Neuherberg, Germany; d Metabolomics and Proteomics Core (CF-MPC), Helmholtz Munich, German Research Center for Environmental Health, Neuherberg, Germany; e Department of Molecular Physiology of Exercise and Nutrition, German Institute of Human Nutrition (DifE) Potsdam-Rehbruecke, Nuthetal, Germany; f Institute of Nutritional Sciences, University of Potsdam, Nuthetal, Germany; g Metabolic Physiology, Institute for Diabetes and Cancer, Helmholtz Munich, German Research Center for Environmental Health, Neuherberg, Germany; h Department of Internal Medicine II, TUM School of Medicine and Health, TUM University Hospital, Technical University of Munich, Munich, Germany; i Division of Neurobiology of Diabetes, TUM School of Medicine and Health, Technical University of Munich, Munich, Germany; j Institute of Experimental Genetics, Helmholtz Munich, German Research Center for Environmental Health, Neuherberg, Germany; k Department of Biochemistry, Yong Loo Lin School of Medicine, National University of Singapore, Singapore; l Institute of Biochemistry, Faculty of Medicine, University of Ljubljana, Ljubljana, Slovenia

## Abstract

The mitochondrial selenoenzyme thioredoxin reductase 2 (TXNRD2) plays a critical role in redox homeostasis and reactive oxygen species **(**ROS) scavenging. While heart-specific deletion of *Txnrd2* in mice resulted in cardiac dysfunction, TXNRD2 function in skeletal muscle, the major component of lean body mass, remains unclear. In human GWAS the *TXNRD2* locus is associated with total lean mass. Here, we show that *Txnrd2* muscle-specific knockout (mTKO) induces a lean phenotype characterized by muscle atrophy and diminished adipose tissues. mTKO mice were resistant to weight gain on standard and high-fat diet. Whole body glucose clearance was increased, and ATP levels in muscle were decreased, suggesting impaired mitochondrial energy production. Transcriptomic and metabolomic analyses revealed alterations in one-carbon metabolism and related pathways. Despite elevated glutathione levels, changes in key factors of cellular detoxification were consistent with compromised antioxidant defence system. In sum, we unravel that *Txnrd2* deficiency in skeletal muscle rewires whole-body energy metabolism through mitochondrial dysfunction and impaired redox capacity.

## Highlights

• Muscle-specific *Txnrd2* deletion (mTKO) causes muscle atrophy and fat loss.
• mTKO mice resist weight gain on standard and high-fat diets.
• *Txnrd2* loss lowers muscle ATP and disrupts mitochondrial function.
• Omics analyses reveal altered one-carbon metabolism and antioxidant defence.
• *Txnrd2* deficiency modifies redox balance and energy metabolism and.

## Graphical abstract

**Figure:**
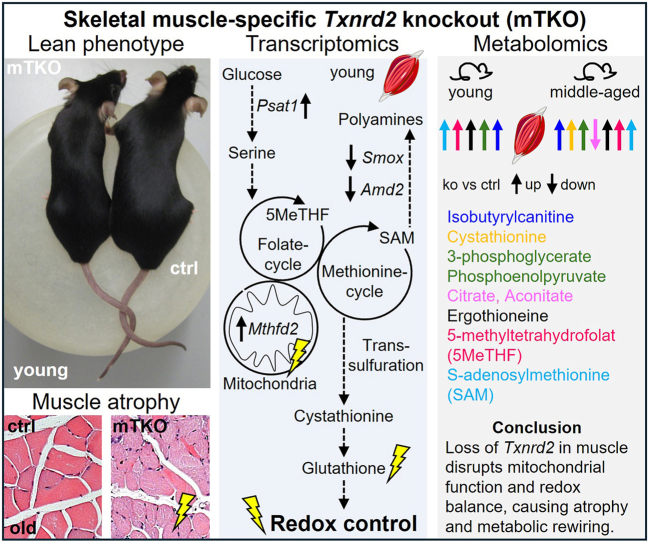

## 1 Introduction

Skeletal muscle (SKM) constitutes approximately 40% of body mass, and plays critical roles in movement, posture, and respiration. Beyond these mechanical roles, SKM is a key metabolic organ involved in systemic lipid, glucose, and protein metabolism [1]. Thus, any change in substrate utilization or energy expenditure in SKM impacts whole-body energy homeostasis and body composition.

While mitochondria generate over 90% of ATP required by SKM during rest and prolonged, moderate-intensity exercise [2], they are also highly susceptible to oxidative damage by continuous production of reactive oxygen species (ROS). A variety of antioxidant enzymes defend against oxidative damage [3], including thioredoxin reductase 2 (TXNRD2) a mitochondrial selenoprotein [4], and one of three major thioredoxin reductase isoenzymes (TXNRDs) in mammals [5]. TXNRDs reduce the disulfide in the active site of thioredoxin (TXN), thus maintaining the pool of reduced and active TXN. TXNRDs are Nicotinamide Adenine Dinucleotide Phosphate (NADPH)-dependent oxidoreductases that transfer electrons to TXN, enabling redox cycling and the maintenance of cellular defence mechanisms against oxidative stress [5,6]. These redox processes also influence multiple signalling pathways via protein-protein interactions [7]. In addition to TXNRD2, glutathione reductase (GSR), found in both mitochondria and the cytosol [8,9], is another key disulfide-reducing enzyme that uses NADPH to convert oxidized glutathione (GSSG) back into its reduced form (GSH), thereby contributing to detoxification and redox homeostasis [6].

Global *Txnrd2* knockout in mice is embryonic lethal, while heterozygous mice are viable [10]. Mice with heart-specific ablation of *Txnrd2* developed a contractile dysfunction along with disassembled mitochondrial structure and functionality [11]. In humans, heterozygous mutations in *TXNRD2* lead to dilated cardiomyopathy [12], while several other studies have found associations to other heart diseases [[13], [14], [15]]. A homozygous stop-gain mutation in a Kashmiri family was associated with familial glucocorticoid deficiency [16], and *TXNRD2* variants are also implicated in primary open-angle glaucoma [[17], [18], [19]]. These observations highlight the relevance of *TXNRD2* in human physiology and diseases.

Body weight differences have a strong genetic component. GWAS still identify an increasing number of loci associated with body composition traits and advances in high-throughput DNA sequencing lead to the identification of loci/genes that are less common and/or have smaller effects [20]. One such GWAS with more than 267,000 participants identified an association of the *TXNRD2* locus with total lean mass in 12 anthropometric measurements related to body size and fat distribution [21,22]. However, to date, the physiological role of *Txnrd2* in skeletal muscle, the major determinant of lean mass, remains largely unexplored. Accordingly, we used a classical reverse genetic strategy to evaluate the molecular impact of skeletal muscle-specific deletion of *Txnrd2* (mTKO) on body composition and whole-body metabolism, muscle and mitochondrial function and alterations in cellular and whole-body metabolism in young and aged mice.

Our results demonstrate that disrupting mitochondrial redox homeostasis in SKM has profound effects on muscle metabolism and overall energy balance. The mTKO mouse model offers a valuable platform to study the systemic consequences of impaired mitochondrial function. It is a powerful model to study the physiological consequences of disturbed mitochondrial redox equilibrium on muscle physiology and body weight control.

## 2 Material and methods

### 2.1 Animal models

Mice were bred and maintained at the Core Facility - Laboratory Animal Services (CF-LAS) at Helmholtz Munich, Germany. They were group-housed with a 12/12‐h light/dark cycle under specific pathogen-free conditions in individually ventilated cages (GM 500, Tecniplast) at room temperature (20 - 24 °C), on commercial wood fiber bedding with additional nesting material, a standardized mouse diet (1314, Altromin Spezialfutter GmbH, Lage, Germany) or a 60% high-fat diet (C1090-60, Altromin), and drinking water *ad libitum*. All animals were bred entirely in house and backcrossed to a C57BL/6 N background. Animal experiments were conducted in accordance with the European Convention for the Protection of Vertebrate Animals used for Experimental and Other Scientific Purposes (No. 123, Strasbourg, France, 1985) and EU Directive 2010/63/EU. Experiments were approved by the Danish Animal Experimental Inspectorate (License No. 2015‐15‐0201‐00477) and performed in compliance with the German animal welfare legislation with approval from the state ethics committee and Government of Upper Bavaria (Az. 55.2-1-54-2532-9-2014, Az. 55.2-2532.Vet_02-18-167, Az. 55.2.1.54-2532‐94‐12). Mice with lox-P-site (fl) containing (floxed) *Txnrd2* alleles (B6.Cg-*Txnrd2*^*tm1Marc*^) [10] were mated to mice expressing CRE recombinase (Cre) under the control of the skeletal muscle-specific human *ACTA1* (actin, alpha 1) promoter (B6.Tg(ACTA1-cre)79Jme/J) [23] to obtain muscle-specific *Txnrd2* knockout mice (B6.*Txnrd2*^*tm1Marc*^Tg(ACTA1-cre)79Jme/CF-LAS), in short mTKO or *Txnrd2*^*fl/fl*^*;* ACTA1-cre^+/Cre^. Littermates were preferred as controls (ctrl, *Txnrd2*^*fl/fl*^). Cre transgenic mice (+/Cre) were bred in a separate colony and used with wildtype (wt) littermates (+/+) as controls (ctrls) as indicated. PCR detection of “wildtype” (+) or “fl” alleles was as described [10]. Primers for “+” and “Cre” alleles were as follows: Cre1: 5′-ACC AGC CAG CTA TCA ACT CG-3′, Cre2: 5′-TTA CAT TGG TCC AGC CAC C -3′, pCtrl1: 5′-CTA GGC CAC AGA ATT GAA AGA TCT-3′, pCtrl2: 5′-GTA GGT GGA AAT TCT AGC ATC ATC C-3’. A ∼200bp band indicated Cre, a ∼350bp band served as positive control. Gel-electrophoresis was performed using QIAxcel® advanced system (Qiagen GmbH, Germany).

### 2.2 Construction of targeting vector and generation of *Txnrd2* overexpression mouse model *Gt(ROSA)26Sortm1.1*(Txnrd2-Ires-LacZ) (Txnrd2Tg)

*Txnrd2* overexpression mouse model was generated as described [24]. In short, to generate the R26-RSR-Txnrd2-IresLacZ knock-in allele, a targeting vector was constructed using a multi-step cloning strategy based on the plasmid pROSA26–1 (RosaRFA), which carries the homology arms required for integration into intron 1 of the Rosa26 locus. The construction of the targeting vector was carried out by standard cloning procedures. All constructs were verified by diagnostic restriction digestion and sequencing.

The Txnrd2 coding sequence was derived from pMC442-Txnrd2-Ires-puro (gift from M. Conrad, Helmholtz Munich) by *Eco*RI/*Sna*BI digestion and ligated blunt-end 5’ of Ires into the multiple-cloning-site (MCS) of vector pEntr-MCS-IresLacZ. The resulting pEntr-Txnrd2-IresLacZ intermediate was then subcloned via *Asc*I/*Aat*II digestion into pEntr-RSR-MCS, yielding pEntr-RSR-Txnrd2-IresLacZ.

The final targeting vector pROSA-RSR-Txnrd2-IresLacZ was assembled by a Gateway Clonase reaction between the entry clone pEntr-RSR-Txnrd2-IresLacZ and the RosaRFA destination vector. The targeting vector contains a rox-flanked transcriptional and translational stop cassette (roxSTOProx, RSR) with a PGK-neomycin resistance cassette 5′ of the Txnrd2-IresLacZ expression cassette, followed by a diphtheria toxin (DTA) negative selection marker outside the long homology arm for selection. The functionality of the targeting vector was validated in cell culture.

We electroporated the *Pac*I-linearized targeting vector into IDG3.2 embryonic stem cells and selected appropriately targeted clones with 140 μg/ml Geneticin. Homologous recombined clones were identified by PCR. Single-copy insertion of the transgene was confirmed by real-time PCR. Three confirmed clones were injected into BDF1-blastocysts and transferred into CD1 recipients. Germline transmission was achieved in 3 out of 3 clones harbouring the targeted allele. Genotyping was carried out using PCR and Txnrd2RSRTg animals were backcrossed to C57BL/6 N.

To generate the *Txnrd2* overexpression mouse model (Txnrd2Tg) we deleted the rox flanked stop cassette in Txnrd2RSRTg animals by crossing them with ubiquitous CAGGs-Dre deleter mice. *Txnrd2* overexpression was confirmed by β-Galactosidase staining of embryos at E14.5.

### 2.3 Survival analysis

From our experimental colony, we have followed all offspring (207 mice in total) from 26 litters for a lifespan study. Animals used for mating were excluded. Animals displaying sings of pain or illness were euthanized and noted as humane endpoint. Mice that were found dead without previous signs of pain or illness were noted as natural deaths. Survival analysis was made using GraphPad Prism 9. Humane endpoints and natural deaths were assigned as death events, while mice removed for experiments or due to barbering were censored and thus are included in the calculation of percent survival until removal.

### 2.4 Body composition and indirect calorimetry

Fat mass and lean mass were measured via Nuclear Magnetic Resonance (NMR) technology (EchoMRI, Houston, TX, USA). Energy expenditure, locomotor activity and food intake were analyzed by a combined indirect calorimetry system (TSE System, Bad Homburg, Germany), as described [25].

### 2.5 Glucose tolerance tests (GTT)

For the GTT, mice were fasted for 6 h 1 h after the onset of the light phase. Subsequently, mice were injected intraperitoneally with 2 g glucose per kg body weight (25% wt/vol d-glucose in 0.9% wt/vol saline). Tail blood glucose levels (mg/dl) were measured using a handheld glucometer (Freestyle lite, Abbott, The Netherlands) before (0 min) and at 15, 30, 60 and 120 min after injection.

### 2.6 Maximal running capacity

Maximal running capacity was conducted as previously described [26]. Briefly, mice were acclimated to treadmill running on three separate days, running for 10 min at 0.17 m/s on a 0° incline. The maximal running capacity test was conducted three days after the last acclimation session and was performed on a 10° incline, beginning with a 5-min warm-up at 0.17 m/s, followed by an increase in speed by 0.02 m/s every minute until exhaustion. The investigator determining exhaustion was blinded to the genotypes.

### 2.7 Ex vivo muscle contraction

Ex vivo muscle contractions of isolated soleus (SOL) and extensor digitorum longus EDL muscles were performed as previously described [27]. Briefly, soleus and EDL muscles were isolated and suspended in incubation chambers containing Krebs Ringer Henseleit (KRH) buffer. Muscles were pre-incubated for 10 min after which platinum electrodes were placed centrally and on both sides of the muscle to induce contraction, using 1-s trains (100 Hz, 0.2 ms, ∼30-40 V) repeated every 15 s for 10 min, while recording the force over time.

### 2.8 Grip strength test

To measure the combined strength of forelimbs and hindlimbs *in vivo*, the mice were placed on a horizontal grid connected to a force sensor (Sauter FK10, 10 N/0,005 N, Kern & Sohn GmbH, Balingen-Frommern, Germany). While holding the mice gently at the base of the tail, they naturally grasp the grid and try to escape. The force is automatically recorded when the mouse tries to move forward until it releases the grid. For each mouse the grip strength was determined three times in a row without a break in between. All tests were performed by the same person.

### 2.9 RNA purification

Total RNA was extracted from muscle tissue stabilized in RNAprotect buffer according to the “Purification of total RNA from animal and human tissue” protocol of the RNeasy Micro Kit (QIAGEN, Hilden, Germany). In brief, the muscle tissue was stored in RNAlater and shipped on dry ice. After thawing and removing the RNAlater, the samples were homogenized in RLT buffer using Precellys CK14 ceramic beads (2 cycles of 20 s at 6500 rpm; 15 s break). Next ethanol was added, and the samples were applied to RNeasy MinElute spin columns followed by an on-column DNase digestion and several wash steps. Finally, total RNA was eluted in 14 μl of nuclease free water. Purity and integrity of the RNA was assessed on the Agilent 2100 Bioanalyzer with the RNA 6000 Nano LabChip reagent set (Agilent, Palo Alto, CA, USA).

### 2.10 GeneChip microarray assay

Sample preparation for microarray hybridization was carried out as described in the Affymetrix GeneChip WT PLUS Reagent Kit User Manual (Affymetrix, Inc., Santa Clara, CA, USA). In brief, 200 ng of total RNA was used to generate double-stranded cDNA. 12 μg of subsequently synthesized cRNA was purified and reverse transcribed into sense-strand (ss) cDNA, incorporating unnatural dUTP residues. Purified ss cDNA was fragmented using a combination of uracil DNA glycosylase (UDG) and apurinic/apyrimidinic endonuclease 1 (APE 1) followed by a terminal labeling with biotin. 3,8 μg fragmented and labeled ss cDNA were hybridized to Affymetrix Clariom S mouse arrays for 16 h at 45 °C in a GeneChip hybridization oven 640. Affymetrix Clariom™ S Mouse Arrays measure gene level expression for ∼20,000 well annotated genes. Because the array includes only constitutive exon probes, it does not allow quantification of alternative splicing. Hybridized arrays were washed and stained in an Affymetrix Fluidics Station FS450, and the fluorescent signals were measured with an Affymetrix GeneChip Scanner 3000 7G. Fluidics and scan functions were controlled by Affymetrix GeneChip Command Console v4.1.3 software. Sample processing and analysis was performed at an Affymetrix Service Provider and Core Facility, “KFB - Center of Excellence for Fluorescent Bioanalytics” (www.kfb-regensburg.de, Regensburg, Germany).

### 2.11 Microarray data analysis

Summarized probe set signals in log2 scale were calculated by using the SST-RMA (1) algorithm with the Affymetrix GeneChip Expression Console v1.4 Software. After exporting into Microsoft Excel, average signal values, comparison fold changes and significance P values were calculated. Probe sets with a fold change above 2.0 fold and a student's *t*-test P value lower than 0.05 were considered as significantly regulated [28].

### 2.12 Plasma and tissue collection for metabolomics

Male mice, aged 8-9 weeks (young) and 27-29 weeks (middle-aged), were sacrificed with carbon dioxide, dipped in 80% ethanol, blotted and tissue samples from musculus gastrocnemius (GTN) were taken in parallel for targeted (20-50 mg) and non-targeted (30-100 mg) analysis. PBS-rinsed samples were blotted, weighed and placed into pre-cooled homogenization tubes containing ceramic beads with a diameter of 1.4 mm (Precellys24 Homogenization Kit, CK14, PeqLab Biotechnology, Erlangen, Germany). All samples were snap-frozen on dry-ice and stored at −80 °C.

### 2.13 Metabolite Quantification

Targeted metabolomics measurements were performed using liquid chromatography- and flow injection-electrospray ionization-tandem mass spectrometry (LC- and FIA-ESI-MS/MS) and the Absolute*IDQ*™ p180 Kit (BIOCRATES Life Sciences AG, Innsbruck, Austria). The assay allows simultaneous quantification of 188 metabolites out of plasma or tissue homogenate. The complete assay procedures as well as the tissue extraction have been previously published [29]. In brief, tissue homogenates were always prepared freshly as follows: Frozen murine muscle tissue samples were weighted into homogenization tubes with ceramic beads (1.4 mm). For metabolite extraction, to each 1 mg of frozen murine muscle tissue,3 μL of a 4 °C cooled mixture of ethanol/phosphate buffer (85/15 v/v) were added. Tissue samples were homogenized using a Precellys24 homogenizer (PEQLAB Biotechnology GmbH, Erlangen, Germany) 3 x for 30 s at 5500 rpm and −4 °C, with 30 s pause intervals to ensure constant temperature, followed by centrifugation at 10,000×*g* for 5 min. Subsequently, 10 μL of the supernatants were placed into the cavities of the 96-well filter plate of the p180 assay. Samples were dried in a nitrogen stream for 30 min. Amino acids and biogenic amines in the samples were derivatized with an excess of 5 % phenylisothiocyanate for 20 min and dried in a nitrogen stream. Samples were extracted for 30 min at RT with 300 μL methanol containing 5 mM ammonium acetate. The LC run was performed using an Agilent XDB-C18 column (3 × 100 mm, 3.5 μm). Sample handling was performed by a Hamilton Microlab STAR™ robot (Hamilton Bonaduz AG, Bonaduz, Switzerland) and an Ultravap nitrogen evaporator (Porvair Sciences, Leatherhead, U.K.), beside standard laboratory equipment. Mass spectrometric analyses were done on an API 4000 triple quadrupole system (SCIEX Deutschland GmbH, Darmstadt, Germany) equipped with a 1260 Series HPLC (Agilent Technologies Deutschland GmbH, Böblingen, Germany) and an HTC-xc PAL auto sampler (CTC Analytics, Zwingen, Switzerland) controlled by the software Analyst 1.6.2. For the LC-part, compounds were identified and quantified based on scheduled multiple reaction monitoring measurements (sMRM), for the FIA-part on MRM. Data evaluation for quantification of metabolite concentrations and quality assessment were performed with the software MultiQuant 3.0.1 (SCIEX) and the Met*IDQ*™ software package, which is an integral part of the Absolute*IDQ*™ Kit. Metabolite concentrations were calculated using internal standards and reported either in pmol/mg for wet tissue or μmol/L (μM) for tissue homogenate.

### 2.14 Analyses with concentrations below the limit of detection (LOD)

LOD is defined as three times of the median of three empty samples (Zero Samples), what is accepted praxis for FIA and HPLC analyses and used in the p180 assay. Metabolites were included in the analysis only if at least 7 out of 15 replicates (≥46.7 %) showed concentrations above the LOD; for these metabolites, values for samples at or below the LOD were edited to 0.

### 2.15 Non-targeted metabolomics

Samples were stored frozen in a −80 °C freezer until processed for extraction. For the extraction process, each tissue sample was first homogenized with water, at a ratio of 15 μl of water per 1 mg GTN. 100 μL of the resulting homogenate was transferred into a 2 mL 96 deep-well plate for further extraction process.

To extract metabolites and to precipitate the protein, 500 μL methanol extraction solvent containing recovery standard compounds was added to each 100 μL of tissue homogenates. Supernatants were aliquoted in 96 well microtiter plates, dried under nitrogen stream and stored at −80 °C until the LC-MS/MS measurements were performed.

In addition to the study samples, a pooled extract comprising all study samples was prepared and aliquoted (100 μL per well) for further extraction in a 2 mL 96 deep well plate. Furthermore, 100 μL of a human reference plasma sample was extracted independently. These samples served as control replicates throughout the study to assess process variability. Besides these samples, 100 μLof water was extracted independently. These samples served as blank controls. Before LC-MS/MS measurement in positive ion mode, the samples were reconstituted with 50 μL 0.1% formic acid in water. The samples analyzed in negative ion mode were reconstituted with 50 μL 6.5 mM ammonium bicarbonate in water (pH 8.0). Reconstitution solvents for both ionization modes contained internal standards that allowed monitoring of instrument performance and served as retention markers. Reverse phase (RP)/UPLC-MS/MS analysis was performed on a Q-Exactive high resolution/accurate mass spectrometer (Thermo Fisher Scientific GmbH, Dreieich, Germany) coupled with a Waters Acquity UPLC system (Waters GmbH, Eschborn, Germany). Two separate columns (2.1 × 100 mm Waters BEH C18, 1.7-mm particle-size) were used for acidic (solvent A: 0.1% formic acid in water, solvent B: 0.1% formic acid in methanol) and for basic (solvent A: 6.5-mM ammonium bicarbonate (pH 8.0), solvent B: 6.5 mM ammonium bicarbonate in 95% methanol) mobile phase conditions, optimized for positive and negative electrospray ionization, respectively. After injection of the sample extracts, the columns were developed with a gradient of 99.5% A to 98% B over an 11-min run time at a flow rate of 0.35 mL/min. The eluent flow was directly routed through the electrospray ionization source of the Q-Exactive mass spectrometer. The full MS scan was performed from 80 to 1000 m/z and alternated between MS and MS/MS scans using a dynamic exclusion technique, which enables a wide range of metabolite coverage.

Metabolites were annotated by curation of the UPLC-MS/MS data against proprietary Metabolon's chemical database library (Metabolon, Inc., Durham, NC, USA) based on retention index, precursor mass and MS/MS spectra. The metabolites were assigned to cellular pathways based on PubChem, KEGG, and the Human Metabolome Database.

### 2.16 Protein extraction and western blotting

One whole musculus gastrocnemius (GTN) muscle was homogenized in 1 ml NET [30](20 mM HEPES pH 7.9, 1.5 mM MgCl, 0.2 mM EDTA, 20% Glycerol, 1% Triton X100, 1 mM DTT, 0.05 mM PMSF, phosphatase inhibitor (Roche PhosSTOP™, Merck, Darmstadt, Germany), protease inhibitor (Roche cOmpleteTM Protease Inhibitor Cocktail, Merck, Darmstadt, Germany) using Precellys CK14 ceramic beads (Precellys24 Homogenization Kit, CK14, PeqLab Biotechnology, Erlangen, Germany) (3 cycles of 30 s at 5500 rpm between 0 °C and 4 °C; 30 s break). Homogenates were centrifuged (10.000g, 15 min, 4 °C) and supernatants stored at −20 °C until use. Protein concentration was measured using the Bradford method and, unless otherwise stated, 30 μg protein were separated by SDS-PAGE before transfer to a nitrocellulose membrane (Amersham™ Protran™ 0.45 μm, Merck, Darmstadt, Germany). After blocking (tris‐buffered saline with 0.1% tween 20 and 5% nonfat milk, 1 h at room temperature) probed with the primary antibodies, in the following dilutions: 1:10 to 1:500 anti‐TXNRD2 (clone 1C4 as described earlier [11]),1:500 to 1:3.000 anti‐MTHFD2 (12270-1-AP, Proteintech Germany GmbH, Planegg-Martinsried, Germany), 1:3.000 anti‐SLC7A5 (bioss bs-10125R, VWR International GmbH, Darmstadt, Germany), 1:2.000 anti‐PSAT1 (10501-1-AP, Proteintech), 1:1.000 anti‐PAO (sc-166185, Santa Cruz Biotechnology, Inc., Heidelberg, Germany), 1:5.000 anti‐STBD1 (11842-1-AP, Proteintech), 1:500 to 1:3.000 anti‐ASNS (14681-1-AP, Proteintech), 1:1.000 anti‐ALAS1 (16200-1-AP, Proteintech), 1:1000 anti‐ANGPTL4 (18374-1-AP, Proteintech), 1:5.000 anti‐DBT (12451-1-AP, Proteintech), 1:500 to 1.000 anti‐GPIHBP1 (NB110-41537, Novus biologicals, Bio-Techne GmbH, 65205 Wiesbaden Nordenstadt, Germany), 1:1.000 to 1.3000 anti-LPL (MABS1270, Merck, Darmstadt, Germany), 1:500 to 1:1.000 anti‐ETFA (12262-1-AP, Proteintech), 1:1.000 anti‐ACOT2 (15633-1-AP, Proteintech). As loading control anti‐ACTIN (23660-1-AP, Proteintech), anti‐GAPDH (Sigma G9295, Sigma G9545, Merk, Darmstadt, Germany, or 12262-1-AP, Proteintech), or anti‐TUBA1B (11224-1-AP, Proteintech) were used. Secondary antibodies were HRP-conjugated anti-mouse (W4021, Promega GmbH, Walldorf, Germany), anti-rabbit (W401B, Promega GmbH, Walldorf, Germany, or SA00001-2, Proteintech) or anti-rat (7077S, Cell signaling). Finally, peroxidase activity was detected by using ECL Detection Reagent (Amersham Cytiva ECL™ Prime Western Blotting System, Merck, Darmstadt, Germany) on Hyperfilm ECL high performance chemiluminescence films (Amersham Cytiva Hyperfilm™ ECL™, Merck, Darmstadt, Germany).

### 2.17 Histology, immunohistochemistry and image analyses

Tissue samples were fixed in 4% paraformaldehyde for 24 h on 4 °C, dehydrated using a graded ethanol series and embedded in paraffin. Sections were cut by microtome (Microtom HM 355 S, MICROM International GmbH, Walldorf, Germany) and put on glass slides. The dewaxed slides were stained, re-dehydrated and mounted on coverslips (ROTI®Histokitt II,

Carl Roth, Karlsruhe, Germany) and viewed in a microscope (Zeiss Mikroskop Axioplan 2 Imaging, Carl Zeiss Microscopy GmbH, Oberkochen, Germany), pictures were obtained using a Zeiss Axiocam MRc camera and visualized with the Axio Vision SE64 Rel.4.9 software (Carl Zeiss Microscopy GmbH, Oberkochen, Germany). 3 μm thick sections from GTN and liver were stained with hematoxylin and eosin (H&E) or Masson's trichrome. Sections were stained using anti-LPL (1:50, MABS 1270 Merck, Darmstadt, Germany), and MTHFD2 (1:200, 12270-1-AP, Proteintech Germany GmbH, Planegg-Martinsried, Germany) and anti-ATP5A1 (1:20, 66037-1-IG, Proteintech). Level of LPL staining was scored as follows: no staining (0), discrete (1) if only focal staining was present, moderate (2) if multifocal staining was present, and intense (3) if diffuse staining was present.

### 2.18 Quantification and scoring of changes in skeletal muscle

Muscle morphological changes of four cohorts of mice were assessed by an observer blinded to sex, age, and genotype by visual inspection of H&E-stained paraffin cross-sections from GTN at 200x magnification. The following four cohorts were analyzed: (1) ctrl versus mTKO males at three different ages, (2) wt versus Cre/+ males at two different ages, (3) ctrl versus mTKO females at three different ages, and (4) ctrl versus mTKO versus mTKOTg females. Each of the 19 groups included at least six individuals. Body and GTN weight were determined upon dissection. For each individual, one tissue section was evaluated as follows: Three pictures were taken from each section to quantify myofiber size by determining the cross-sectional area (CSA) using Image J analysis software (version 1.46) (National Institutes of Health) [31] via segmentation and particle analysis. The threshold for particle analysis was set between a minimum area of 200 μm^2^ and a maximum limit of 4000 μm^2^ to exclude small particles that do not represent muscle fibers and large particles that are muscle fibers sticking together. Low contrast between cell boundaries and their surroundings resulted in multiple particles being recognized as one. In such cases, cell boundaries were sometimes manually drawn. After locating and isolating fibers in an image, the size of each fiber was assigned as the number of pixels it occupied, with 392 pixels corresponding to 200 μm. Fiber sizes were written to output files and summary statistics were generated for each image. Acquired data was used to calculate mean CSA. Histograms comparing the distribution of myofibers were created from this data. As histology was performed on PFA fixed, dehydrated, paraffin embedded muscle, which can introduce shrinkage and interstitial spacing artifacts, we interpret absolute fiber size with caution and rely on relative comparisons. The number of degenerating (swollen, hypereosinophilic) fibers was counted for the entire cross section, and the number of central nucleated fibers (CNF) was counted for three visual fields. The degree of myoblast abundance, necrosis, and cell infiltration was assessed by visual inspection (200x magnification) on a scale of 0 to 3 as follows: normal/none (0), moderate and focal only (1), severe and multifocal (2), and very strong and diffuse (3).

### 2.19 Whole mount X-gal staining for β-galactosidase activity

To detect *LacZ* expression in embryos and in muscle from adult mice carrying the Txnrd2Tg allele, we performed classical X-gal (5-bromo-4-chloro-3-indolyl-β-d-galactopyranoside) staining with standard protocols using potassium ferricyanide and potassium ferrocyanide. Specimens were pre-fixed for 90 min and post-fixed overnight each in 4% PFA.

### 2.20 Mitochondrial Respiration

GTN was dissected from both hind legs and placed into ice-cold buffer A (0.1 M KCl, 0.05 M Tris/HCl, 2 mM EGTA). Mitochondria were isolated using differential centrifugation as previously described with modifications [11]. Briefly, GTN was isolated and minced with a scalpel after removing accessible fat and connective tissue. Afterwards, buffer A was replaced by buffer B (0.5% BSA, 5 mM MgCl_2_, 1 mM ATP, 245.7 U/100 ml Protease Typ VIII added to buffer A; 1 ml per 100 mg tissue) and samples stirred gently on ice for 2 min. Then tissue was homogenized using 6 strokes in a glass/glass homogenizer (Ace Glass 8325-16, 15 ml). After adding 10 ml of buffer A, homogenates were cleared from debris and nuclei via centrifugation (2.000g, 10 min, 4 °C), mitochondria were pelleted (10.000g, 10 min, 4 °C), washed twice (10.000g, 3 min, 4 °C), and resuspended in 120 μl buffer A. Protein concentrations were determined with Bradford assay. Age matched knockout and control mitochondria were always processed and measured side-by-side on the same experimental day, eliminating day-to-day variability as a confounding factor. O_2_-consumption was assessed at 37 °C using a Clark-type electrode (Oxygraph, Hansatech Instruments Ltd, H S Laborbedarf, Reutlingen, Germany) as described [11] (150 μg mitochondira in 750 μl respiration buffer: 0.14 mol/L mannitol, 0.05 mol/L sucrose, 5 mmol/L MgCl2, 0.25 mol/L EDTA, 0.01 mol/L phosphate, 2 mmol/L Tris‐HCl, pH 7.4). The respiratory substrates (8 mmol/L succinate, 12,5 μM rotenone, 300 μM ADP, 2 μM oligomycin and 2 μM FCCP) were added as indicated.

### 2.21 ATP measurement

Upon dissection, 70-150 mg GTN were washed in PBS and underwent Phenol-Chloroform extraction as previously published [32]. Briefly, tissue was homogenized in ice-cold 3 ml Phenol-TE (P4557, Sigma, Merck, Darmstadt, Germany), using an Ultra-Turrax (IKA T18 basic). 1 ml of the homogenate was added to 200 μl Chloroform and 150 μl DEPC water, vortexed and centrifuged for 5 min at 10.000 g on 4 °C. Supernatant was stored on −20 °C until measurements were performed. Samples were diluted 1:200 in DEPC water, followed by a 1:5 dilution in ATP detection sample buffer. With 10 μl thereof an ATP assay (ATP Detection Assay Kit-Luminescence, Cayman 700410, Biomol GmbH, Hamburg Germany) was performed using a microplate reader according to the manufacturer's instructions.

### 2.22 GSH measurement

Upon dissection, approx. 100 mg GTN were washed, blotted and stored on −80 °C until measurements were performed. Tissue was homogenized in 10 ml/g MES buffer using Precellys CK14 ceramic beads (Precellys24 Homogenization Kit, CK14, PEQLAB Biotechnology GmbH, Erlangen, Germany) (3 cycles of 30 s at 5500 rpm between 0 °C and 4 °C; 30 s break). Homogenates were centrifuged (10.000g, 15 min, 4 °C), protein concentration of supernatant was measured using Bradford method and after deproteinization GSH assay (Glutathione Assay Kit, Cayman 703002, Biomol GmbH, Hamburg Germany) was performed according to the manufacturer's instructions.

### 2.23 NADPH measurement

About 20 mg frozen GTN (stored at 80 °C) was homogenized in 400 μl Lysis buffer (kit component) using Precellys CK14 ceramic beads (Precellys24 Homogenization Kit, CK14, PEQLAB Biotechnology GmbH, Erlangen, Germany) (3 cycles of 30 s at 5500 rpm between 0 °C and 4 °C; 30 s break). Homogenates were centrifuged (2.500 rpm, 10 min, 4 °C), protein concentration of supernatant was measured using Bradford method, and 50 μl of the supernatant were used for NADPH assay (NADP+/NADPH Assay Amplite, AAT Bioquest, 15276) according to the manufacturer's instructions.

### 2.24 Blood chemistry

Mice were sacrificed, and blood samples taken by puncturing the vena cava. Blood was collected in EDTA-containing centrifuge tubes and centrifuged at 4 °C and 3.000 rpm for 10 min. Cholesterol and triglycerides levels were analyzed from plasma using the scil Reflovet® Plus (Antech Diagnostics Germany GmbH, Viernheim, Germany).

### 2.25 Statistical analysis

Statistical analyses were performed using GraphPad Prism (V.9 or higher), Microsoft Excel, or SPSS. Two groups were compared by using two-tailed unpaired Student's t-test. Combined indirect calorimetry measurements were assessed by Analysis of Co-Variance (ANCOVA), using body weight as predictive covariate for total EE (F(1,12) = 7.302, p = 0.019) and light-phase EE (F(1,12) = 23,840, p < 0.001) but not dark phase EE (F(1,12) = 0.483, p = 0.499). Results are shown as mean ± SD values. Bar- and dot plots, volcano plots and heatmaps were constructed using GraphPad Prism or Metaboanalyst [33]. n = number of animals per group or experiments as indicated. Statistical significance was defined as P < 0.05 (∗), P < 0.01 (∗∗), or P < 0.001 (∗∗∗).

### 2.26 Data availability

Source data are provided with this paper. The raw data from the Affymetrix gene expression study have been deposited in the Gene Expression Omnibus (GEO) under accession number GSE310521. Processed data from the gene expression study, are provided in Supplementary Table 1. Metabolomics data underlying the figures are provided in Supplementary Tables 2–5.

## 3 Results

### 3.1 Generation of tissue-specific *Txnrd2* knockout mice

**Fig. 1:**
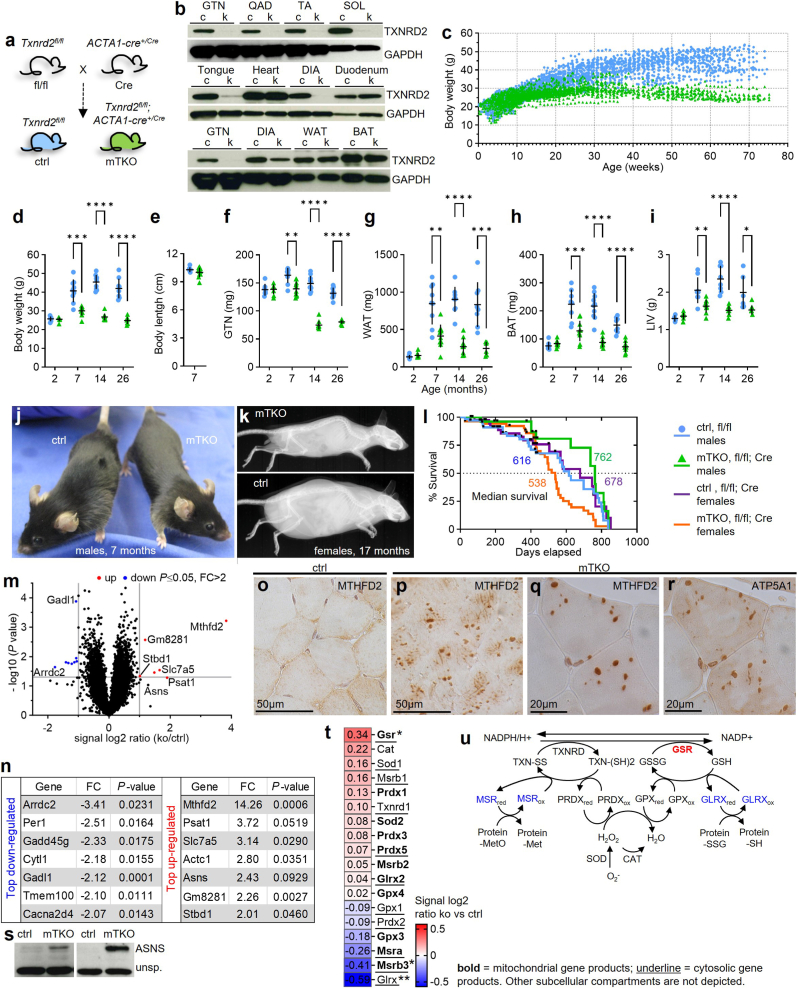
***Txnrd2* inactivation in skeletal muscle induces a lean phenotype.** **a**, Generation of skeletal muscle-specific *Txnrd2* knockout (ko) mice (mTKO). **b**, Validation of *Txnrd2* ko in skeletal muscle, but no other major tissue type, by Western blot analysis. 30 μg protein were loaded except 20 μg for WAT and 10 μg for BAT. Expression varies with connective tissue in mTKO diaphragm. **c**, Body weight growth curves of male mice. Each point represents the weight of an individual mouse. **d-i,** Body weight and length (naso-anal length) and weight of tissues collected from male mice of different ages as indicated. **j,** Representative image of a 7-month-old mTKO male compared to a control. **k**, Radiographs of female mice revealing an increase in spine curvature in aged mTKOs. **l**, Kaplan-Meier survival curves of ctrl and mTKO male and female mice (n = 69 male and 39 female for ctrl, n = 43 male and 56 female for mTKO). **m**, Volcano plot for the affymetrix microarray gene expression analysis of GTN tissue from mTKO mice compared to ctrls. Differentially expressed genes (DEGs), defined by a *P* value ≤ 0.05 and a fold change (FC) > 2 are shown as red (upregulated) or blue (downregulated) dots. **n**, Top regulated genes defined by a *P* value ≤ 0.05 and a fold change (FC) > 2. **o-r**, Representative images of immunohistochemistry using anti-MTHFD2 (**o-q**) and anti-ATP5A1(**r**) specific antibodies on GTN paraffin cross-sections. (**o**) and (**p**) n = 3 mice per genotype. (**r**) Consecutive section of (**q**). **s**, Protein abundance of ASNS. **t**, Heatmap showing gene expression changes of selected genes related to redox regulation and antioxidant defence as determined by microarray analysis (Supplementary Table 1). Genes are presented in decreasing order of signal log2 ratio. A signal log2 ratio of 1.0 represents an increase in transcript level by a fold-change of two (2 FC) and −1.0 reflects a decrease by a fold-change of two (−2 FC). Transcript levels are displayed in red when upregulated or in blue when downregulated. n = 5 for ctrl and mTKO. **u**, Redox systems [6], representing enzymatic activities shared by multiple isoenzymes. Compartment-specific isoforms are indicated in Fig. 1t. Subcellular localization may vary between tissues, the assignments reflect canonical annotations rather than muscle-specific data. Reduced proteins carry the subscript ‘red’ and oxidized forms ‘ox’. Red colour indicates increased and blue decreased transcript levels as shown in (**t**). Only significant alterations are shown in respective colours. Modified from Ref. [35]. **d-i**, Results are mean ± SD. *P* values by unpaired two-tailed *t*-test. **b** and **d-i**, GTN, musculus (m) gastrocnemius; QAD, m. quadriceps femoris; TA, m. tibialis anterior; SOL, m. soleus; DIA, diaphragm; WAT, inguinal white adipose tissue; BAT, intrascapular brown adipose tissue; LIV, liver. Asterisks ∗ indicate statistical significance of mTKO (fl/fl; Cre) versus ctrl (fl/fl) of the same age. Statistical significance was defined as *P* ≤ 0.05 (∗), *P* ≤ 0.01 (∗∗) or *P* ≤ 0.001 (∗∗∗). Genotypes as indicated.

To investigate the role of skeletal muscle TXNRD2 we deleted *Txnrd2* selectively in myofibers using *ACTA1* promoter-driven Cre recombinase, active in fast- and slow-twitch fibers but inactive in satellite cells [23,34]. *Txnrd2*^*fl/fl*^ mice crossed with *ACTA1-Cre*^*CRE/+*^ (Cre) produced control (ctrl, *Txnrd2*^*fl/fl*^*ACTA1-Cre*^*+/+*^) and knockout (mTKO, *Txnrd2*^*fl/fl*^*; ACTA1-Cre*^*+/Cre*^) offspring (Fig. 1a). Western blots confirmed TXNRD2 loss in gastrocnemius (GTN), quadriceps (QAD), tibialis anterior (TA), and soleus (SOL) of mTKO mice and decreased expression in tongue and diaphragm. TXNRD2 was detected in duodenum, heart, white (WAT) and brown adipose (BAT), confirming muscle-specific deletion except in heart (Fig. 1b).

MTKO mice, born at normal ratios, stopped gaining weight at ∼10 weeks (males) and ∼30 weeks (females) (Fig. 1c, Supplementary Fig. S1h–j). Aged mTKO mice showed decreased GTN, WAT, BAT, and liver (LIV) weights with unchanged body length (Fig. 1d–j, Supplementary Fig. S1a–g). Male mice carrying the *ACTA1-Cre*^*CRE/+*^ (Cre/+) alone showed no difference in body weight or muscle and liver mass compared to wt controls (+/+) (Supplementary Fig. S1k–p).

With age, mTKO mice developed kyphosis, indicating muscle weakness (Fig. 1k, Supplementary Fig. S1q and r). Median survival was lower in mTKO females, because they reached termination criteria/humane endpoints earlier due to low body weight, but surprisingly, was higher in mTKO males, presumably from lack of obesity compared to ctrl males (Fig. 1l). Despite tissue weight loss, morphology and histology of fat appeared unchanged (Supplementary Fig.S1s-u).

These results indicate that *Txnrd2*-deficient muscle causes systemic alterations leading to a lean phenotype.

### 3.2 *Txnrd2* ko increases *Mthfd2* expression along with changes in antioxidant genes

To uncover how muscle-specific loss of *Txnrd2* is involved in muscle atrophy, we focused on gene expression signatures in skeletal muscle. Microarrays of GTN muscles from 2-month-old mTKO mice showed robust upregulation of methylenetetrahydrofolate dehydrogenase 2 (*Mthfd2*) (FC = 14.3, P ≤ 0.05), a nucleus encoded gene localized to mitochondria (Fig. 1m and n; Supplementary Table 1). *Mthfd2* is typically active in developing tissues but is known to be induced in adult skeletal muscle in response to mitochondrial stress [36]. MTHFD2 staining was detected in mTKO GTN but not in controls (Fig. 1o–q). ATP5A1 confirmed mitochondrial localization of the signal (Fig. 1r). Increased GTN expression of ASNS, an enzyme that regulates asparagine biosynthesis and amino acid homeostasis, was confirmed also at the protein level (Fig. 1m,n,s).

Mitochondria contain their own TXN and glutathione systems. Glutathione reductases (GSRs) catalyze the regeneration of GSH from GSSG, utilizing NADPH. Glutaredoxins (GLRXs) and methionine sulfoxide reductases (MSRs) are enzymes involved in the regeneration of oxidized proteins [6,37]. In mTKO muscle, *Gsr* transcript level was elevated, indicating enhanced GSH regeneration, while *Glrx* and *Msrb3* transcripts were decreased (Fig. 1t and u). These changes indicate complex regulation across cellular compartments. *Glrx* encodes the cytosolic glutaredoxin [38], while *Glrx2*, whose gene products localize to mitochondria and the cytosol [39], was unchanged. *Gsr* gene products localize to the cytosol and mitochondria [8], and MSRB3 isoforms localize to the endoplasmic reticulum and mitochondria [40].

As *Gsr* is an established target of nuclear factor erythroid 2 related factor 2 (NRF2) [41], we examined additional NRF2 responsive genes, including *Nqo1*, *Sqstm1/p62*, *Mt1*, *Mt2*, *Srxn1* and GST family members, however, none of these were altered significantly in our transcriptomic dataset (data not shown and Supplementary Table S1)

Together, these transcriptomic changes observed in the mTKO mice already at the age of 2 months indicate compensatory regulation of skeletal muscle metabolism involving proliferation, amino acid metabolism, and redox homeostasis in the absence of *Txnrd2*.

### 3.3 mTKO mice are protected from diet-induced obesity

**Fig. 2:**
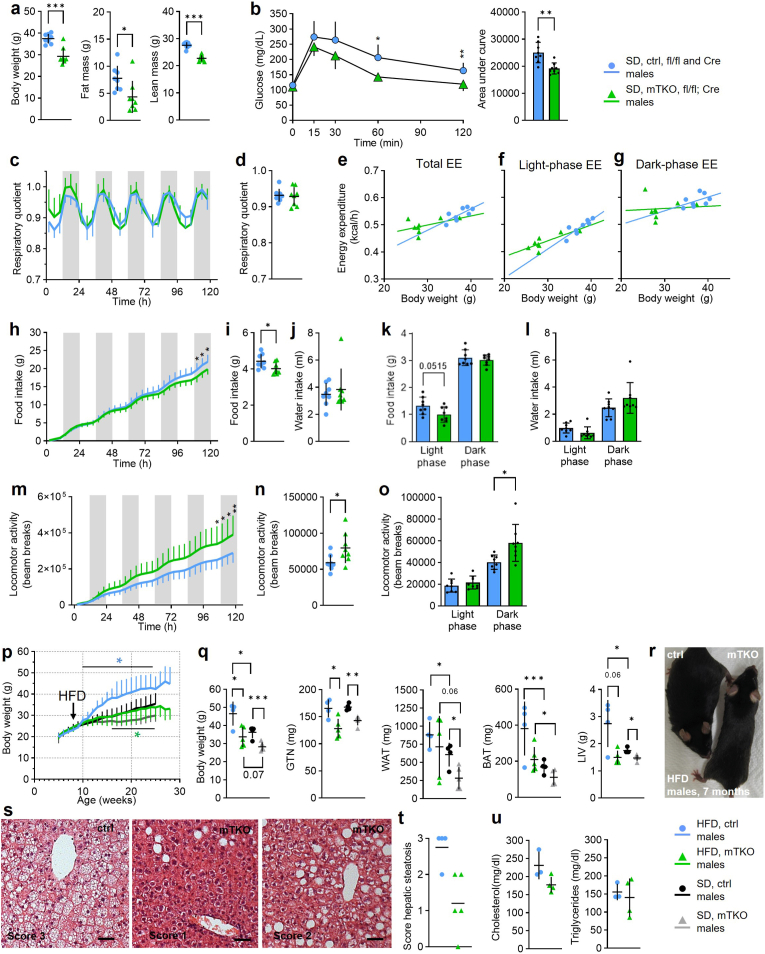
**mTKO male mice are protected from diet-induced obesity.** **a**, Body weight, fat and lean mass of mTKO versus ctrl. **b,** Glucose tolerance test (GTT). **c,** Respiratory exchange ratio (RER) over 5 days, **d,** relative daily RER, **e,** total energy expenditure (EE), **f,** light-phase EE, **g,** dark-phase EE, **h,** cumulative food intake, **i,** average daily food intake, **j**, average daily water intake, **k**, day and night food intake, **l,** day and night water intake, **m,** cumulative locomotor activity, **n,** average daily locomotor activity and **o,** average day and night locomotor activity were assessed in male mTKO mice compared to ctrl in indirect calorimetry chambers. **a**-**o,** n = 8 male mice for both genotypes. **p,** Body weight growth curves of male mice receiving a high-fat diet (HFD) from the age of 8 weeks on, compared to males on standard chow diet (SD) (n = 4-5 for ctrl, n = 4-5 for mTKO on HFD and n = 10-12 for ctrl, n = 8-12 for mTKO on SD). **q,** Body weight and isolated tissue weights of male mice after HFD or SD feeding (n = 4 for ctrl, n = 5 for mTKO on HFD and n = 4 for ctrl, n = 4-5 for mTKO on SD). **r**, Representative images of male mice. **s,** Representative images of H&E-stained liver paraffin-sections from HFD-fed male mice. Scale bar, 50 μm. **t,** Scoring of hepatic steatosis based on H&E-staining of livers form HFD-fed male mice (n = 4 sections form 4 animals for ctrl, n = 5 sections form 5 animals for mTKO). **u**, Blood cholesterol and triglyceride levels after HFD feeding. (n = 3 mice for ctrl, n = 4 mice for mTKO). Genotypes as indicated.

To better understand potential origins of sex-specific differences in growth, we performed metabolic analyses at 8 to 10 months of age, when sex-specific differences in body weight are fully established in males but have only just begun to manifest in females (Supplementary Fig. S1h and j). On standard chow diet (SD) mTKO males had a decreased body weight and lower fat and lean mass compared to controls (Fig. 2a). Intriguingly, glucose clearance in a glucose tolerance test (GTT) was significantly improved (Fig. 2b). Respiratory exchange ratio (RER; Fig. 2c and d) and total (F(1,12) = 0.095, p = 0.763), light- (F(1,12) = 0.064, p = 0.804) and dark-phase (F(1/12) = 0.309,p = 0.588) energy expenditure (EE) values, analyzed by ANCOVA and adjusted for body weight, were comparable between genotypes (Fig. 2e–g). Food and water intake were modestly lower in mTKO males mainly during the light phase (Fig. 2h–l), whereas locomotor activity was increased during the dark phase indicating more explorative behaviour (Fig. 2m–o).

Females on SD likewise showed tendencies towards decreased lean mass and improved glucose clearance, with unchanged RER, EE, intake, and activity (Supplementary Fig. S2a–o). These findings suggest that, under standard chow conditions, modestly reduced food intake and increased activity co-occur with lower body weight in males and may contribute to leanness at this time point, whereas females show no difference in food intake or activity and likewise do not yet display differences in body weight.

High-fat diet (HFD) increased body weight in both male and female mTKO mice, but magnitude of effect remained significantly lower than in HFD-fed controls (Fig. 2p–r; Supplementary Fig. S2p–r). Decreased body weight on HFD aligned with lower GTN, WAT, BAT, and LIV mass (Fig. 2q; Supplementary Fig. S2q). Liver steatosis upon HFD was milder in male mTKOs and minimal in females (Fig. 2s and t; Supplementary Fig.S2s,t). HFD-fed male mTKOs showed trends toward lower cholesterol and triglyceride levels (Fig. 2u), no changes were seen in female mTKOs (Supplementary Fig. S2u). Collectively, our data suggest that mTKO mice maintain lower body weight under both chow and HFD conditions, accompanied by decreased adiposity and milder HFD-induced hepatic alterations, with similar tendencies in males and females albeit differing in magnitude.

### 3.4 *Txnrd2*-deficient muscles show progressive degeneration, atrophy and functional decline

**Fig. 3:**
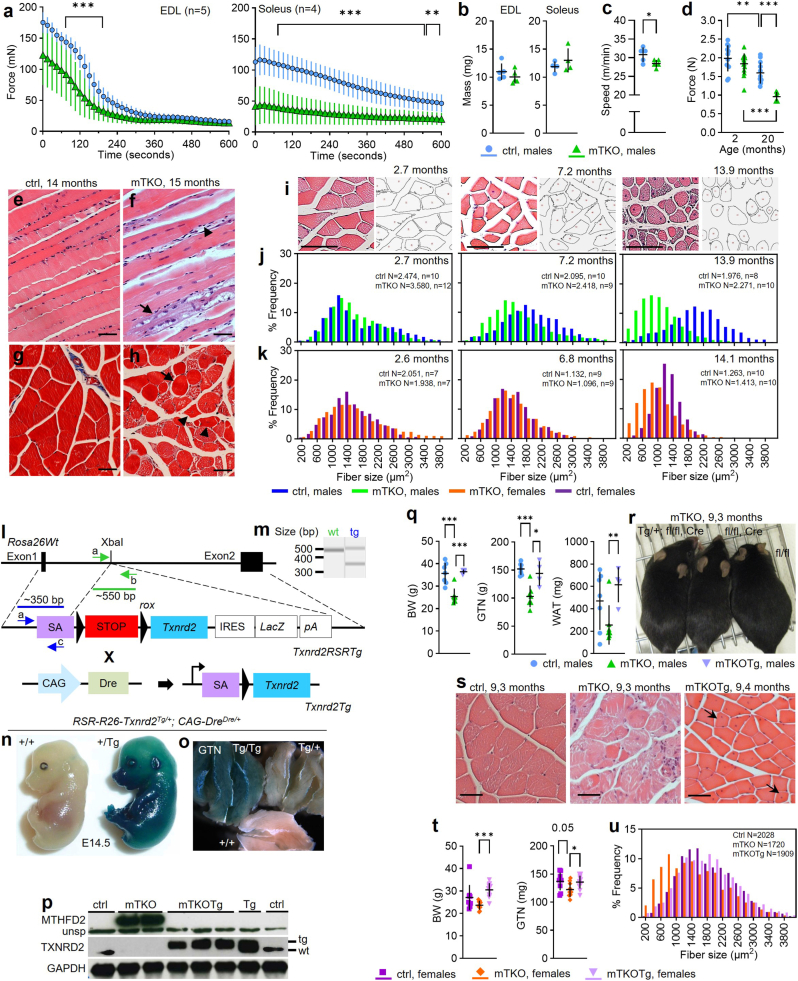
***Txnrd2*-deficient muscle shows atrophy with functional decline.** **a**, Force development recordings during 600 s electrically induced contraction in EDL and soleus muscles (n = 5 for EDL and n = 4 for soleus for each genotype; age: 2 months). **b,** Muscle weight of EDL and soleus used in (**a**). **c,** Maximal running speed (n = 5 for each genotype). **d,** Force determined by grip strength measurement in mice aged 2 (n = 12 animals for ctrls, n = 15 animals for mTKO) and 20 months (n = 12 animals for ctrls, n = 6 animals for mTKO). **e** and **f,** Representative longitudinal sections of paraffin embedded GTN stained with H&E. **g** and **h,** Representative cross-sections stained with Masson's Trichrome. Scale bar, 50 μm. **i**, Representative H&E-stained paraffin GTN sections from male mTKO mice and their corresponding muscle fibers classification maps as they were the basis for evaluation of data shown in (**j**) and (**k**). **j** and **k,** Quantification of myofiber sizes of GTN from mTKO versus ctrl. Histograms showing relative frequency of myofiber size (cross sectional area, CSA) in GTN of (**j**) male and (**k**) female mice. N: number of CSA values of all animals in a group. Scale bar 200 μm. **l**, Schematic of the *Txnrd2* wildtype transgene (*Txnrd2* Tg) knockin strategy and the resulting constitutive overexpression mouse model (mTKOTg). **m**, Genotyping strategy using primers a-c shown in (**l**). **n** and **o**, β-Galactosidase staining of (**n**) whole mount embryos and (**o**) GTN from adult mice. **p-u,** Overexpression study of *Txnrd2* in mTKO background. **p,** Protein abundance of MTHFD2, TXNRD2 and GAPDH. **q,** Body, GTN, and WAT weight of male mice (n = 7 animals for ctrl, n = 7 animals for mTKO, n = 4 animals for mTKOTg; age: 9 months). **r**, Representative images of male mice. **s,** Representative H&E-stained paraffin GTN sections from males. Arrows indicating CNF. Scale bar: 50 μm. **t,** Body and GTN weight of female mice (n = 9 animals for ctrl, n = 9 animals for mTKO, n = 12 animals for mTKOTg; age: 9 months. **u,** Histogram showing relative frequency of myofiber sizes in GTN.

Despite unchanged muscle mass at 2-months, mTKO males showed severely impaired muscle function, including diminished muscle force in EDL and SOL (Fig. 3a and b), lowered maximal running capacity (Fig. 3c), and decreased grip strength, which severely worsened with age (Fig. 3d).

Histology analyses of 14-month-old mTKO GTN muscles revealed fiber degeneration, basophilic fragmentation, and central nucleated fibers (CNFs), indicating past regenerative events (Fig. 3e–h). Around 7 months of age myofiber size distribution in mTKO males showed a significant shift toward smaller fibers, with more severe atrophy by 14 months (Fig. 3i and j). This effect was delayed in mTKO females, with more pronounced differences observed at 14 months (Fig. 3k). Collectively, our findings suggest progressive muscle atrophy in mTKO mice is mainly due to age-related degeneration.

### 3.5 Ectopic TXNRD2 reverts *Txnrd2*-deficiency associated phenotypes

To confirm a direct role of *Txnrd2* knockout in the observed phenotypes, we restored *Txnrd2* in mTKO mice by crossing the wildtype *Txnrd2* transgene (Txnrd2Tg), inserted at the Rosa26 locus and activated by DRE recombinase, into the mTKO background yielding mTKOTg mice (mTKOTg) (Fig. 3l and m). The ROSA26 promoter is active from early embryogenesis and supports broad, constitutive expression across embryonic and adult tissues [42]. LacZ expression in embryos and adult GTN muscles confirmed Tg expression (Fig. 3n and o). Western blots showed ectopic TXNRD2 and absence of MTHFD2 in mTKOTg mice (Fig. 3p). 9-month-old mTKOTg males and females had body and GTN muscle weights similar to controls, while mTKOs showed decreased values (Fig. 3q,r,t). Overall, we noticed fewer signs of degeneration in mTKOTg muscles. However, CNFs persisted (Fig. 3s). On the other hand, mTKOTg females showed normal fiber size distribution (Fig. 3u).

### 3.6 Muscle atrophy is due to the *Txnrd2* ko and independent of cre recombinase

**Fig. 4:**
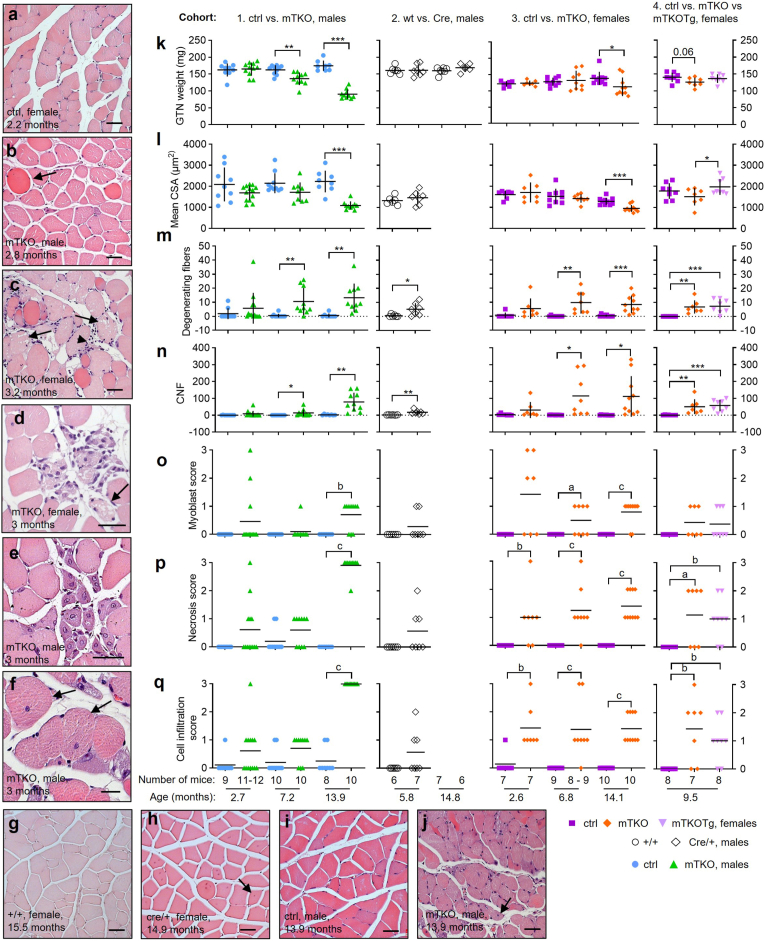
**Muscle atrophy in mTKO mice is independent of CRE-expression.** **a**-**q**, Muscle morphological alterations (**a**-**j**) were quantified and scored (**k**-**q**). **a**-**j,** Exemplary structures of representative cross-sections of paraffin embedded GTN stained with H&E of ctrl (**a, i**), mTKO (**b**-**f**,**j**), +/+ (**g**) and Cre/+ (**h**), female (**a**,**c**,**d**,**g**,**h**) and male (**b**,**e**,**f**,**i**,**j**) mice, showing normal morphology (**a**,**g**,**i**), exhibiting multiple features of degeneration (**b**-**d**,**j**), and features of regeneration (**e**,**f**,**h**). i. e. degenerating (swollen and hypereosinophilic) fibers (**b**, arrow), necrotic (fragmented and vacuolated) fibers (**c**,**d**, arrows), myoblasts (small basophilic myocytes with central nuclei) (**e**), cell infiltration (**c**, arrowhead), centrally nucleated fibers (CNF) (**f**,**h**,**j**, arrows). **k**-**q,** Muscle morphology was quantified and scored for four cohorts of mice comparing ctrl versus mTKO (cohort 1 males and cohort 3 females), +/+ versus Cre-bearing male mice (cohort 2) and ctrl versus mTKO versus mTKOTg female mice (cohort 4) at different ages as indicated. Genotype, sex and age as indicated. **k**, GTN weight, determined upon dissection. **l,** Cross-sectional area (CSA) calculated using data acquired with Image J [31]. **m**, Number of degenerating fibers counted for the whole cross-section. **n**, Number of CNFs counted in three visual fields. **o**-**q,** Level of myoblast abundance (**o**), necrosis (**p**), and cell infiltration (**q**) was determined on a scale from 0 to 3 as discernible by light microscopy. **a**-**g**, Scale bar, 20 μm (**f**), 50 μm (**a**-**c**,**e**,**g**-**j**) and 100 μm (**d**). Expanded interstitial spaces caused by fixation and/or sectioning artifact. **k**-**q,** Each symbol in the graphs represents data or mean from one individual. Data is shown as mean with SD (**k**-**n**) or mean only (**o**-**q**). Statistical significance was evaluated using Student's t-test (**k-n**) or Mann-Whitney-U-Test (**o**-**q**) and was defined as *P* ≤ 0.05 (∗, a), *P* ≤ 0.01 (∗∗, b) or *P* ≤ 0.001 (∗∗∗, c). Asterisks ∗ and letters indicate statistical significance as indicated.

To clarify which morphological abnormalities in mTKO mice result from *Txnrd2*-deficiency, we analyzed GTN histology across four mouse cohorts. Histological differences were compared between fl/fl (ctrl) and fl/fl; Cre (mTKO) males (cohort 1) and females (cohort 3), +/+ and +/Cre males (cohort 2), and fl/fl, fl/fl; Cre, and Tg/+; fl/fl; Cre (mTKOTg) females (cohort 4), aged 3, 7, and 14 months (Fig. 4a–q). GTN weight and CSA declined in aging mTKO mice but remained stable in Cre/+ and ctrl animals, tissue loss was rescued in mTKOTg females (Fig. 4k and l).

These results confirm that *Txnrd2*-deficiency causes atrophy independently of Cre expression. However, degenerating (swollen and hypereosinophilic) fibers (Fig. 4b) and CNFs (Fig. 4f,h,j) appeared also in Cre/+ males and mTKOTg females (Fig. 4m and n), implicating a role of Cre in some damage. Myoblast-rich areas (Fig. 4e) were sporadically seen in young mTKO mice and occasionally in older ones (Fig. 4o). Old mTKO mice exhibited severe degeneration: degenerating fibers, necrosis, and infiltrates (Fig. 4j,m,p,q). Cre/+and mTKOTg muscles also showed degeneration but retained structural integrity (Fig. 3s; Fig. 4h,m,p,q).

These findings attribute degeneration primarily to *Txnrd2*-deficiency, supporting the atrophy phenotype. The presence of CNFs and myoblasts suggests that regeneration is not unique to *Txnrd2*-deficiency related damage but may also result from Cre-induced injury.

### 3.7 Metabolomics identifies alterations in major biochemical pathways

**Fig. 5:**
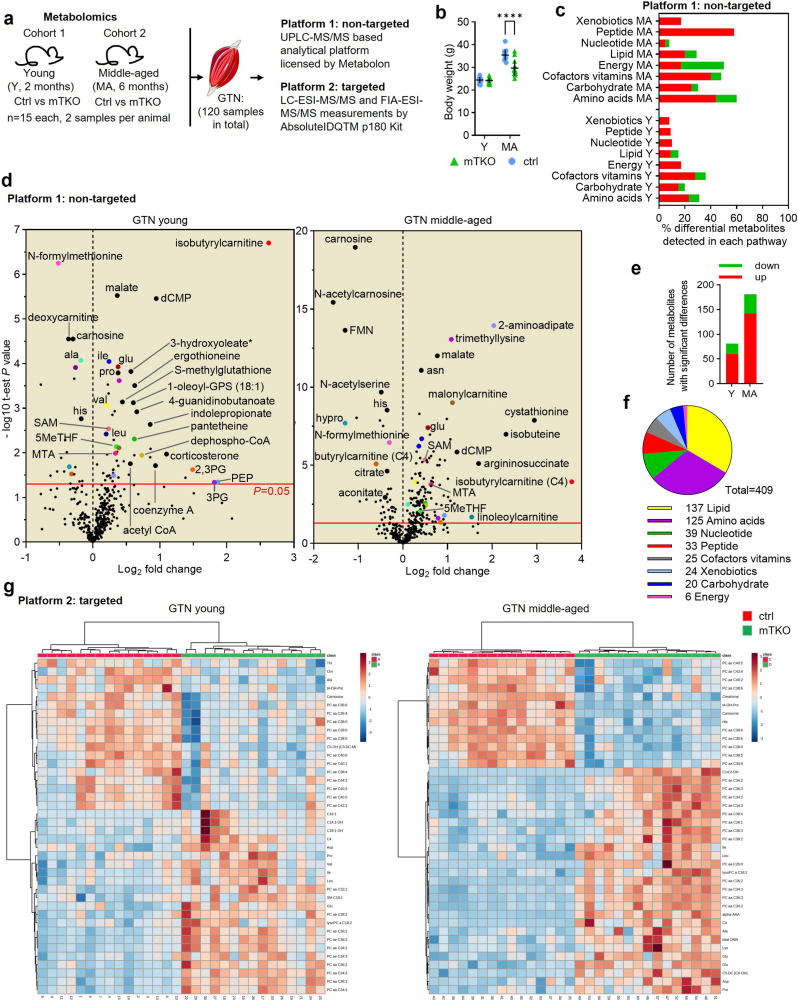
***Txnrd2*-deficiency induces alterations in the metabolome that increase with age.** **a**, Schematic showing experimental design of targeted and non-targeted metabolomic analyses. **b**, Body weight of animals used in (**a**). **c** and **e**, Visualization of differential metabolites as determined by Student's t-test from non-targeted metabolomics using GTN from young (Y) and middle-aged (MA) mice as indicated. Cut-off *P* ≤ 0.05. **c,** Super pathway distribution of differential metabolites. **d**, Volcano plots showing differential metabolites. The x-axis represents log_2_ fold-change of mTKO versus ctrl and the y-axis represents -log_10_ of *P*-values. Colours indicate the same metabolites in both age groups. Ala, alanine; asn, asparagine; ile, isoleucine; glu: glutamate; pro, proline; his, histidine; dCMP, 2′-deoxycytidine 5′-mono-phosphate; SAM, S-adenosylmethionine; 5MeTHF, 5-methyltetrahydrofolate; MTA, 5-methylthioadenosine; CoA, coenzyme A; 2,3 PG, 2,3-diphosphoglycerate; PEP, phosphoenol-pyruvate; 3 PG, 3-phosphoglycerate; FMN, flavin mononucleotide; hypro, *trans*-4-hydroxyproline; HPMA, S-(3-hydroxypropyl)mercapturic acid (HPMA); HMVA, 2-hydroxy-3-methylvalerate. **e,** Number of known metabolites with significant different abundance. **f**, Number of known metabolites per super pathway. **g**, Hierarchical clustering heatmap of the top 50 metabolites with significantly (one-way ANOVA) altered abundance in GTN from mTKO versus ctrl. Data from targeted metabolomics analyzed by MetaboAnalyst 5.0 [33] using Ward's method and Euclidean distance measure. Heatmap colours reflect concentration values. Each row represents one metabolite, and each column one biological sample. Red indicates higher and blue indicates lower metabolite amount. See Supplementary Tables 2 and 3 (c-f) and Supplementary Tables 4 and 5 (g) for raw data.

To unravel metabolic perturbations induced by *Txnrd2*-deficiency in skeletal muscle, we next applied untargeted and targeted metabolomics to GTN samples of 2- and 6-month-old mTKO and control mice (n = 15 per group) (Fig. 5a–g; Supplementary Tables 2–5). Two months of age mark the end of rapid postnatal growth [43], while at six months, mTKO and control muscle weights diverge but are not yet affected by sarcopenia [44].

Untargeted metabolomics identified 409 known metabolites across biochemical pathways, with differential metabolites (P ≤ 0.05) increasing with age (Fig. 5c,e,f; Supplementary Tables 2 and 3). Volcano plots visualized changes in abundance and significance (Fig. 5d). Targeted data revealed genotype-specific clustering of samples using the top 50 differential metabolites (Fig. 5g).

Isobutyrylcarnitine, showing the highest fold-change at both ages, along with changes in other acylcarnitines, suggest impaired mitochondrial beta-oxidation (Fig. 5d; Supplementary Tables 2 and 3). Both platforms consistently showed alterations in amino acids (AAs) and related metabolites. Glutamine, serine, and tyrosine were unchanged. Most AAs were increased in skeletal muscle of 6-month-old mTKO mice. Isoleucine, leucine, valine, glutamate, proline, and tryptophan were elevated at both ages. Histidine and ornithine were less abundant, suggesting early consumption. Alanine was decreased in young and increased in middle-aged mTKO (Supplementary Fig. S3a and b). Increased dipeptides in older mTKOs support muscle catabolism (Supplementary Fig. S3c). These data reveal progressive metabolic disruption with age in *Txnrd2*-deficient muscle, implicating mitochondrial dysfunction and altered protein turnover in the observed atrophy.

### 3.8 *Txnrd2*-deficiency triggers redox perturbations in SKM

**Fig. 6:**
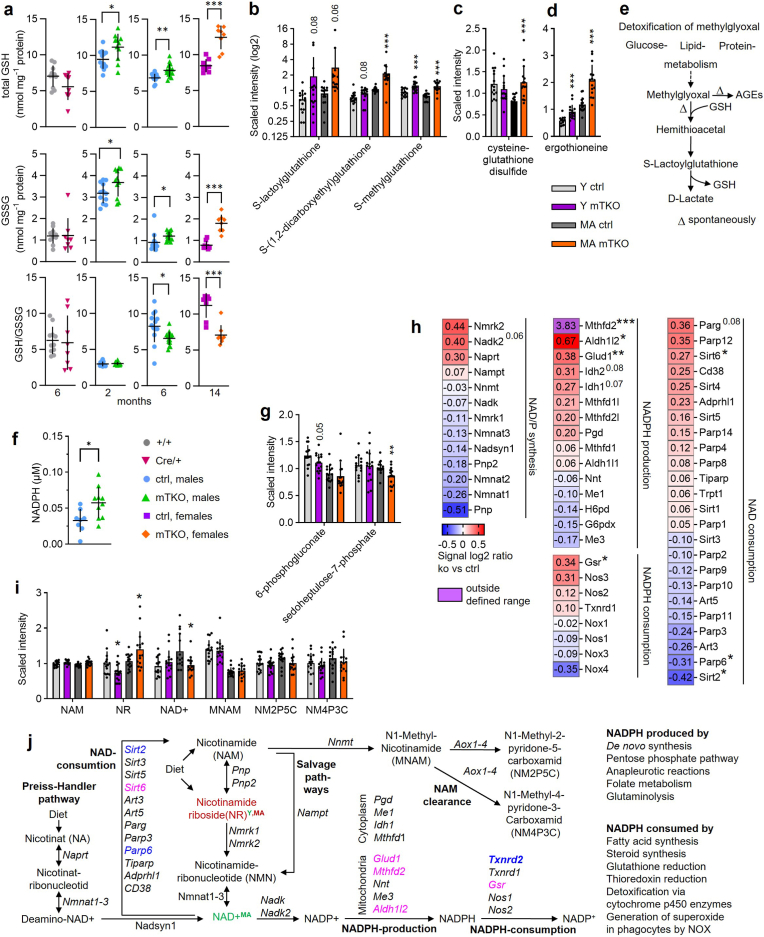
**Redox and antioxidative capacity is altered in mTKO muscle.** **a,** Amount of GSH and GSSG measured in GTN (males) and QUAD (females) tissue, and calculated GSH to GSSG ratio. Age and genotype as indicated. **b**-**d,** Abundance of metabolites as evaluated by non-targeted metabolomics related to (**b**,**c**) glutathione metabolism and (**d**) xenobiotics respectively antioxidative defence. Different y-axes were used to improve visual clarity. **e,** Schematic of methylglyoxal detoxification. **f**, NADPH amount determined in GTN form mTKO versus ctrl from mice at the age of 3 months. **g**, Metabolites related to pentose phosphate pathway as determined by non-targeted metabolomics. **h**, Heatmap showing gene expression changes of selected genes related to NAD/NADPH metabolism as determined by microarray analysis. Genes are presented in decreasing order of signal log2 ratio for each column. A signal log2 ratio of 1.0 represents an increase in transcript level by a fold-change of two (2 FC) and −1.0 reflects a decrease by a fold-change of two (−2 FC). Transcript levels are displayed in red when upregulated or in blue when downregulated. n = 5 for ctrl and mTKO. **i**, Abundance of metabolites related to nicotinamid metabolism as determined by non-targeted metabolomics. **j,** Qualitative changes of metabolite and transcript abundance as determined by metabolomics and transcriptomics mapped onto a schematic of nicotinamid metabolism drawn based on KEGG pathways. Differentially expressed genes (DEGs) are represented by their gene symbols in pink if overexpressed or blue if underexpressed in mTKO muscle compared to ctrl. Only significant alterations are shown in respective colours. Cut off for DEGs: *P* value ≤ 0.05 and a fold change (FC)≥1.2. Metabolites with significantly altered abundance in mTKO muscle compared to ctrls are shown in red (increased) or green (decreased). Time-point of analysis is indicated in superscript, distinguishing tissue analyzed from young (Y) and middle-aged (MA) animals. **b**-**d**,**g**,**i**, Asterisks∗ indicate statistical significance of mTKO versus ctrl of the same age.

The cytosolic and mitochondrial TXN systems, along with the glutathione system, maintain redox homeostasis and can compensate for each other when one pathway is impaired [45]. To assess glutathione alterations due to *Txnrd2*-deficiency, we measured GSH and GSSG in mTKO muscle at 2, 6, and 14 months. Both were elevated, unlike in Cre/+ controls. Status of GSH (GSH/GSSG ratio), a stress marker [46], was decreased at 6 and 14 months. TXNRD2-Tg overexpression normalized glutathione levels (Fig. 6a; Supplementary Fig. S4a).

GSH-related detoxification products, including cysteine-glutathione disulfide, ergothioneine, and S-lactoylglutathione, were increased in mTKO GTN (Fig. 6b–d). Elevated S-lactoylglutathione suggests enhanced methylglyoxal detoxification, a reactive glycolytic byproduct linked to glycation [47] (Fig. 6e). Gamma-glutamyl amino acid abundance also increased (Supplementary Fig. S4b), indicating shifts in glutathione turnover.

NADPH, the reducing agent for TXN and glutathione systems, was elevated in mTKO muscle (Fig. 6f). However, key PPP metabolites, 6-phosphogluconate and sedoheptulose-7-phosphate, were decreased, suggesting altered flux (Fig. 6g). Changes in NADP + precursors, including NAD+ and nicotinamide ribose, were observed (Fig. 6i and j). Transcript levels of *Gsr* and mitochondrial NADPH-generating enzymes were increased, but not those for cytosolic equivalents (Fig. 6h, j; Fig. 1t). These findings indicate disrupted glutathione dynamics, heightened antioxidant demand, and altered NADPH metabolism in *Txnrd2*-deficient muscle.

### 3.9 *Txnrd2*-deficient muscle reveals alterations in one-carbon metabolism

One-carbon (1C) metabolism supports biosynthesis, epigenetics, and redox balance via folate and methionine cycles. Pathways including polyamine synthesis and transsulfuration compete for the limited 1C pool. Serine, a key 1C donor, enters the cycle through folate, producing glycine, and derives from dietary protein or glucose metabolism [48].

**Fig. 7:**
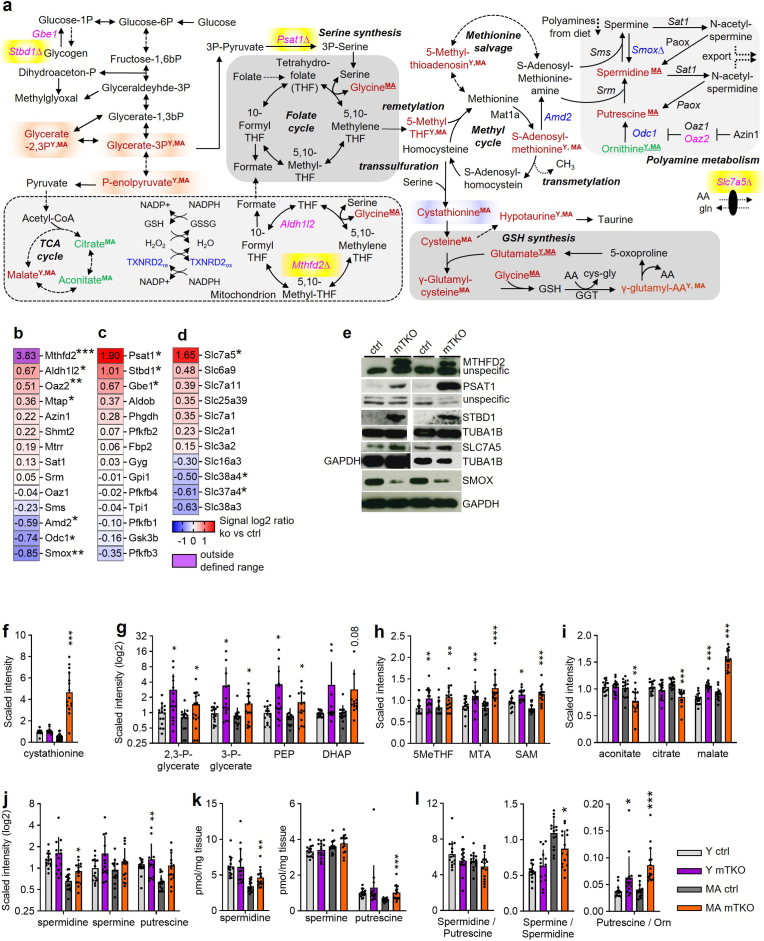
**Alterations in one-carbon metabolism and interconnected pathways in mTKO.** **a**, Qualitative changes of metabolite and transcript abundance mapped onto simplified schematic excerpts of respective KEGG pathways as determined by transcriptomics and metabolomics. Differentially expressed genes (DEGs) are represented by their gene symbols in pink if overexpressed or blue if underexpressed. Only significant alterations are shown in respective colours. Cut off for DEGs: *P* value ≤ 0.05 and a fold change (FC)≥1.2. The four top upregulated genes are highlighted in yellow. A triangle Δ indicates increased (pink) or decreased (blue) protein abundance of respective gene on western blots. Metabolites with significantly altered abundance are shown in red (increased) or green (decreased). Timepoint of analysis is indicated in superscript, distinguishing tissue analyzed from young (Y) and middle-aged (MA) animals. Timepoint is underlined when metabolite was regulated in the same direction, at the same timepoint in targeted and non-targeted metabolomics. **b-d,** Heatmap showing gene expression changes of selected genes related to (**b**) methionine and polyamine metabolism, (**c**) carbohydrate metabolism, and (**d**) solute carrier family, as determined by microarray analysis. Genes are presented in decreasing order of signal log2 ratio for each column. A signal log2 ratio of 1.0 represents an increase in transcript level by a fold change of two (2 FC) and −1.0 reflects a decrease by a fold change of two (−2 FC). Transcript levels are displayed in red when upregulated or in blue when downregulated. n = 5 for ctrl and mTKO. **e,** Protein abundance of MTHFD2, PSAT1, STBD1, SLC7A5 and SMOX. TUBA1B, GAPDH or unspecific bands were used as loading control as indicated. **f**-**l,** Abundance of metabolites as evaluated by (**f-j**) non-targeted, and (**k,l**) targeted metabolomics related to (**f**) transsulfuration pathway and glutathione, (**g**) carbohydrate, (**h**) methionine, (**i**) TCA-cycle, and (**j**,**k**,**l**) polyamine metabolism. (**l**) Ratio of the amounts of the respective polyamines to one another. Note that to increase visual clarity different y-axis are used. **b**-**d**,**f**-**k** Asterisks ∗ indicate statistical significance of mTKO versus ctrl of the same age.

In *Txnrd2*-deficient muscle, *Mthfd2*, part of the mitochondrial folate cycle, was strongly induced (Fig. 7a,b,e; Fig. 1m,n,p,q; Fig. 3p), prompting integration of omics data (Fig. 7a). Transcripts and proteins related to amino acid transport (*Slc7a*), serine synthesis (*Psat1*), glycogen metabolism (*Stbd1*, *Gbe*), and mitochondrial folate metabolism (*Aldh1l2*, *Mthfd2*) were upregulated. Polyamine synthesis genes (*Smox, Odc1*) were downregulated (Fig. 7b–e).

Cystathionine, from the transsulfuration pathway and a glutathione precursor, showed the second highest change in aged mTKO muscle (Fig. 7f). Alterations were observed in glucose, 1C, and polyamine metabolism, as well as in TCA intermediates (Fig. 7g–l). Shifts in polyamine ratios, including spermine to spermidine, may indicate age-related dysfunction [49,50].

These findings link altered 1C metabolism and connected pathways to mTKO muscle pathology.

### 3.10 Mitochondrial dysfunction in *Txnrd2*-deficient muscle

**Fig. 8:**
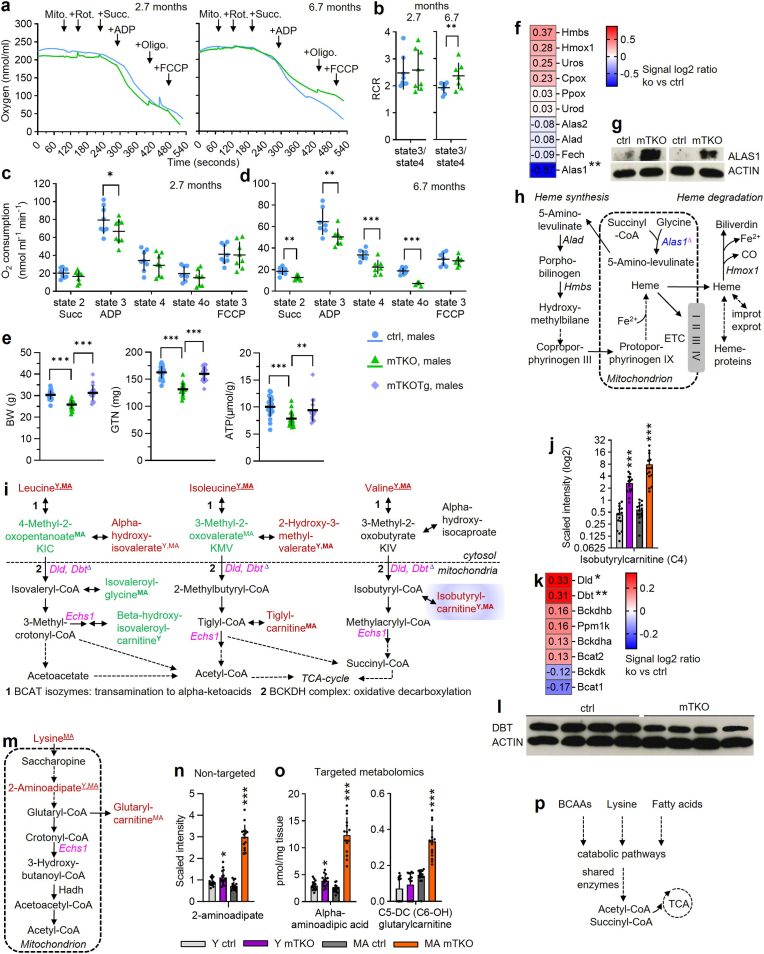
***Txnrd2*-deficiency induces mitochondrial dysfunction in SKM.** **a**, Representative oxygen electrode traces assessed under addition of mitochondria isolated from GTN (Mito.), succinate (Succ.), ADP, and the inhibitors rotenone (Rot.), oligomycin (Oligo.), and FCCP. **b**, Respiratory control ratio (RCR). **c** and **d**, Mean oxygen consumption with (**c**) n = 8 animals each for ctrl and mTKO, and (**d**) n = 7 animals each ctrl and mTKO. **e**, Body weight, GTN weight, and ATP content of GTN muscle from n = 23 ctrl, n = 19 mTKO and n = 18 mTKOTg mice. **f,** Heatmap showing gene expression changes of heme pathway genes. **g,** ALAS1 protein abundance. **h**, Schematic of heme synthesis and degradation based on KEGG pathways. **i** and **m**, Qualitative changes of metabolite and transcript abundance mapped onto schematic excerpts of KEGG valine, leucine and isoleucine (**i**), and lysine (**m**) degradation pathways. **h**,**i**,**m**, Differentially expressed genes (DEGs) are represented by their gene symbols in pink if overexpressed or blue if underexpressed in GTN of young animals. Only significant alterations are shown in respective colours. Cut off for DEGs: *P* value ≤ 0.05 and a fold change (FC)≥1.2. Colours and symbols as in Fig. 7a. Purple highlights isobutyrylcarnitine displaying the highest FC in Y and MA. **j**, Abundance of isobutyrylcarnitine as evaluated by non-targeted metabolomics. **k**, Gene expression changes of BCAA catabolism associated genes. **l**, DBL protein abundance. **n** and **o**, Abundance of 2-aminoadipate (synonym: alpha-aminoadipic acid) and glutarylcarnitine. **p**, BCAAs, lysine and fatty acids share catabolic enzymes. (**f** and **k**), Transcript levels analyzed in GTN from mTKO mice versus ctrl. Genes are presented in decreasing order of signal log2 ratio. Transcript levels are displayed in red when upregulated or in blue when downregulated. n = 5 for ctrl and mTKO. Asterisks ∗ indicate statistical significance of mTKO versus ctrl of the same age or as indicated. Statistical significance was defined as *P* ≤ 0.05 (∗), *P* ≤ 0.01 (∗∗) or *P* ≤ 0.001 (∗∗∗). BCAA, branched-chain amino acid; BCAT, branched-chain aminotransferase isozymes; BCKDH, branched-chain a-keto acid dehydrogenase; TCA, tricarboxylic acid.

To assess mitochondrial-specific effects [51] of the mTKO phenotype, we isolated muscle mitochondria and measured oxygen consumption after sequential additions of rotenone, succinate (state 2), ADP (state 3), oligomycin (state 4o), and FCCP (state 3_FCCP_) (Fig. 8a). Normalized mitochondrial protein yield was comparable between genotypes (Supplementary Fig. S5a), indicating similar recovery during isolation. Middle-aged mTKO mitochondria showed increased RCR (Fig. 8b). In young mTKO mice, state 3 respiration was diminished (Fig. 8c), and in middle-aged mTKOs, all states declined (Fig. 8d). FCCP-stimulated respiration was not altered in mTKO compared with control mitochondria at both timepoints tested (Fig. 8c and d). As a technical limitation, we note that FCCP-driven rates did not exceed state 3 respiration. ATP content was lowered in mTKO muscle and restored with Txnrd2Tg (Fig. 8e).

Omics data confirmed impaired mitochondrial function (Fig. 8f–o). In young mTKOs, *Alas1* transcript decreased (Fig. 8f) while ALAS1 protein increased (Fig. 8g), reflecting regulatory adjustments. ALAS1, essential for heme synthesis (Fig. 8h), is tightly regulated to avoid toxic heme. Heme is critical for respiratory chain complexes. However, major gene changes, among genes encoding components of the respiratory chain, were limited to *ND2*, encoding a subunit of complex I, and *Uqcrfs1*, which encodes the Rieske iron-sulfur protein of complex III. (Supplementary Fig. S5b).

### 3.11 *Txnrd2*-deficiency impairs BCAA and lysine metabolism

Branched-chain amino acid (BCAA) catabolism is regulated by cytosolic BCAT1, mitochondrial BCAT2, and the BCKDH complex. BCAT transaminates BCAAs into BCKAs, which BCKDH irreversibly decarboxylates to form short-chain acyl-CoAs in mitochondria [52]. In mTKO muscle, BCAA levels were elevated at both tested time points (Fig. 8i; Supplementary Fig. S3a and b). Breakdown product levels varied (Fig. 8i and j; Supplementary Fig. S5c). Transcripts of BCKDH subunits *Dld* and *Dbt* were upregulated (Fig. 8k), but the amount of DBT protein was lower compared to ctrls (Fig. 8l).

Lysine is catabolized via saccharopine in mitochondria, producing 2-aminoadipate, then acetyl-CoA [53] (Fig. 8m). mTKO muscle showed 2-aminoadipate accumulation in both targeted and untargeted metabolomics (Fig. 8n and o). Glutarylcarnitine, a marker of glutaric acidemia type I [54], was increased in middle-aged mTKO (Fig. 8o). Accumulation of BCAA and lysine intermediates is plausible, as degradation shares enzymes like ECHS1 and HADH [53] (Fig. 8p).

In summary, mTKO muscle exhibits mitochondrial dysfunction, evident in decreased respiration and ATP, altered ALAS1 expression, and impaired BCAA and lysine metabolism.

### 3.12 Altered lipid metabolism in *Txnrd2*-deficient muscle

**Fig. 9:**
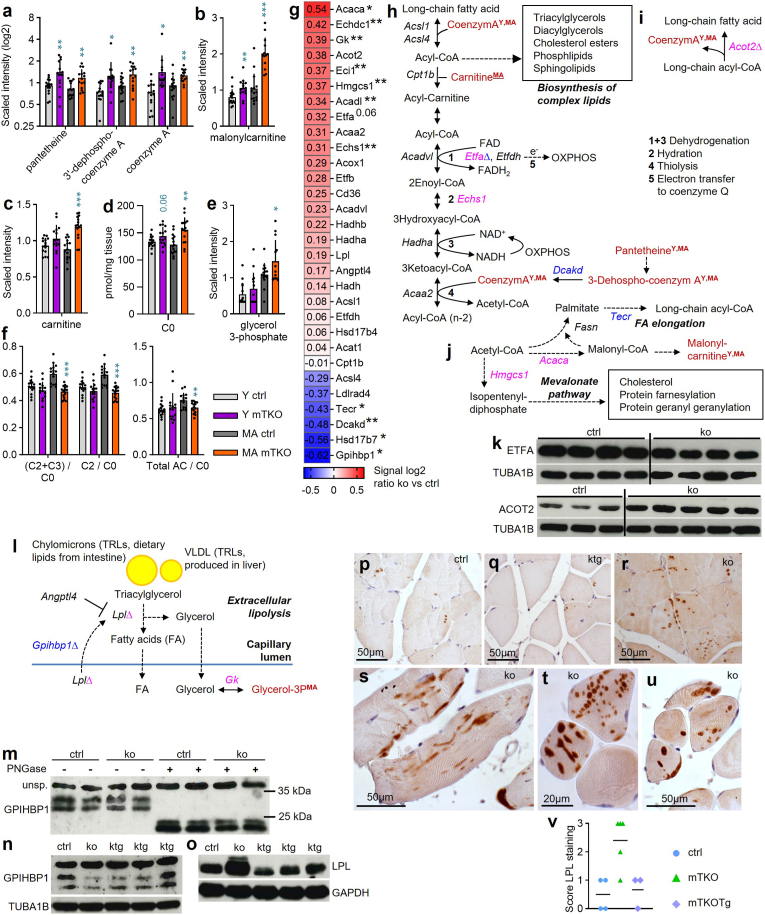
**Lipid metabolism is disturbed in muscle from mTKO mice.** **a**-**e**, Abundance of metabolites associated with (**a**) pantothenate and Coenzyme A, (**b,c,d,e**) carnitine and glycerol metabolism as evaluated by (**a**,**b**,**c**,**e**) non-targeted, and (**d,f**) targeted metabolomics. Asterisks ∗ indicate statistical significance of mTKO versus ctrl of the same age. Different y-axes were used to improve visual clarity. **g,** Heatmap showing gene expression changes of selected genes related to lipid metabolism as determined by microarray analysis. Genes are presented in decreasing order of signal log2 ratio. **h**-**j**,**l**, Schematic representations illustrating qualitatively altered metabolite abundance and gene expression changes in (**h**) degradation, and (**j**) elongation of FAs, and (**i**) hydrolysis of long-chain fatty acyl-CoA into free FA and CoA, and (**l**) lipolysis of triglycerides and fatty acid (FA) uptake. Colours and symbols as in Fig. 7a. **k,** Protein abundance of ETFA and ACOT2. TUBA1B was used as loading control. **m**-**o**, Protein abundance of (**m**,**n**) GPIHBP1, and (**o**) LPL. GAPDH and TUBA1B were used as loading controls. **p**-**u,** Representative images of immunohistochemistry using anti-LPL antibody on GTN paraffin sections from n = 4 mice for ctrl, n = 5 for mTKO and n = 3 for mTKOTg. **v,** Quantification of anti-LPL immunoreactivity. CoA, CoenzymA; OXPHOS, oxidative phosphorylation; TCA, tricarboxylic acid; VLDL, very low-density lipoproteins; TRL, triglyceride-rich lipoprotein.

Lipid metabolism reflects mitochondrial function [55]. In mTKO muscle, CoA, pantetheine, 3′-dephosphoCoA (Fig. 9a), and malonylcarnitine (Fig. 9b) were elevated. Middle-aged mice showed increased carnitine (Fig. 9c) and free carnitine (C0) (Fig. 9d), glycerol-3-phosphate (Fig. 9e), and long-chain fatty acids (Supplementary Fig. S6a). Lysophospholipids, acyl-carnitines, and fatty acids were differentially altered (Supplementary Fig. S6b–f). Ratios of acylcarnitines to C0, beta-oxidation markers, and lipid fractions also changed (Fig. 9f; Supplementary Fig. S7a). Saturated glycerophosphocholines and plasmalogens (total PCae) decreased (Supplementary Figs. S7a,b,c).

Transcriptome data revealed altered expression of lipid metabolism genes: extracellular lipolysis (*Gpihbp1*), beta-oxidation (*Eci, Echs1*), electron transfer (*Etfa*), and synthesis (*Acaca, Hmgcs1*) (Fig. 9g). ETFA and ACOT2 protein changes confirmed these findings (Fig. 9h–k) as their functions lie in transfer of electrons from several mitochondrial flavoproteins and activity of acyl-CoA thioesterase that hydrolyzes CoA esters to the corresponding free acid and CoA, respectively [56,57]. GPIHBP1 was downregulated. It transports LPL into capillaries and its loss causes chylomicronemia syndrome [[58], [59], [60]]. LPL hydrolyzes triglycerides in SKM and adipose tissue, releasing FFAs. Its expression and secretion respond to nutritional signals (Fig. 9l).

PNGaseF treatment confirmed GPIHBP1 signal identity and its lowered abundance in mTKO (Fig. 9m), which normalized in mTKOTg (Fig. 9n). LPL increased in mTKO but not mTKOTg (Fig. 9o). Immunohistochemistry showed variable LPL accumulation in mTKO but minimal in ctrl and mTKOTg (Fig. 9p–v). These results reflect lipid metabolism alterations are linked to mitochondrial dysfunction.

## 4 Discussion

The *in vivo* roles of thioredoxin reductases (TXNRDs), particularly under physiological stress such as aging or metabolic challenges, remain incompletely understood. Here, we demonstrate that *Txnrd2* is critical for maintaining muscle mass and systemic energy homeostasis in aging mice.

Specifically, we reveal that the loss of *Txnrd2* impairs redox homeostasis and induces muscle-specific perturbations of amino acid metabolism, including elevated dipeptide and increased total amino acid levels in aged mTKO muscle. Consistent with an enhanced proteolysis, the mTKO mice exhibited muscle weakness that preceded overt atrophy and correlated with mitochondrial dysfunction, detectable as early as approximately 10 weeks of age. In general, mTKO mice displayed a frail phenotype that was characterized by a profound lack of fat depots, increased locomotor activity, modestly diminished feeding, improved glucose tolerance, and a resistance to high-fat diet-induced obesity and hepatic steatosis.

In humans, the *TXNRD2* locus was associated with lean body mass, but the corresponding rs529822645-T variant was located within a non-coding region with unknown effects on TXNRD2 expression [21,22]. Our data demonstrate, that *Txnrd2* is an important component of lean mass physiology, primarily driven by its role in skeletal muscle metabolism and function.

*TXNRD2* mutations are linked to human disease: homozygous mutations cause familial glucocorticoid deficiency without cardiomyopathy [16], while heterozygous variants associate with cardiomyopathies and may act via pleiotropic or modifying pathways [12,13,15]. Indeed, a rare case has been reported of an 18-year-old female diagnosed with advanced dilated cardiomyopathy positive for two heterozygous gene variants of uncertain significance, one in the *DES* (desmin) gene and one in *TXNRD2* [13]. These observations underscore the importance of understanding *Txnrd2* function *in vivo* as it may have direct implications for human health.

Most notably, *Txnrd2* deficient mice showed severe signs of muscle weakness consistent with biochemical perturbations such as redox imbalance with elevated NADPH, GSH, and ergothioneine levels and upregulated *Gsr* and decreased *Msrb3* and *Glrx* expression, pointing to compensatory shifts in redox control.

*Gsr* is an established target of NRF2 [41]. NRF2 is activated by oxidative stress or toxicity through inhibition of KEAP1-mediated degradation and subsequent induction of antioxidant response element (ARE)-regulated genes. Additional NRF2 responsive genes, including *Nqo1*, *Sqstm1/p62*, *Mt1*, *Mt2*, *Srxn1* and GST family members, were likewise unchanged in our transcriptomic dataset. Taken together, this indicates that no coordinated activation of the broader NRF2 transcriptional program occurred in this model. Loss of certain redox-active enzymes, such as TXNRD1, can trigger NRF2 activation in other tissues [61], and NRF2 activation can be threshold dependent with distinct biological consequences [62], suggesting that NRF2 activation is not an obligatory consequence of all mitochondrial or redox-related perturbations and may be threshold dependent.

Loss of TXNRD2 did not induce compensatory upregulation of other thioredoxin reductase isoforms: *Txnrd1*, encoding the cytosolic isoform TXNRD1 [5,63], and *Txnrd3*, encoding the testis-enriched isoform TXNRD3 [5], remained unchanged in mTKO muscle at the transcript level. Instead, transcriptional adjustments occurred within the glutathione-dependent redox network: *Gsr* transcripts, whose products localize to both cytosol and mitochondria, were increased, whereas *Glrx* (*Glrx1*), encoding the primarily cytosolic glutaredoxin-1 [63], and *Msrb3* [37], which produces isoforms residing in the ER and mitochondria, showed lower expression. Together with shifts in GSH-linked metabolites, these transcriptional changes indicate that neither parallel TXNRD isoforms nor the glutathione system fully compensate for the loss of mitochondrial TXNRD2, consistent with persistent redox stress rather than effective functional substitution.

We observed *Mthfd2* induction at an age when overt structural degeneration in mTKO muscle was not detected. This temporal dissociation suggests that activation of mitochondrial 1C metabolism may arise independently of measurable morphological decline. At ∼2-3 months, transcriptomic and proteomic analyses confirm *Mthfd2* upregulation while muscle mass and fiber size distributions remained comparable to controls. Such induction is consistent with previous findings linking *Mthfd2* expression to mitochondrial stress signaling in muscle [36]. These observations raise the possibility that *Mthfd2* associated metabolic remodeling contributes to early adaptive processes in *Txnrd2* deficient muscle, a hypothesis that warrants further investigation in future studies.

Upregulated levels of MTHFD2, a mitochondrial enzyme in 1C metabolism, suggested altered folate-driven NADPH production. This is consistent with mitochondrial dysfunction models in mouse and humans that show reprogrammed 1C metabolism, increased serine-driven transsulfuration, and glutathione synthesis in response to impaired oxidative phosphorylation, suggesting a common adaptive process [[64], [65], [66], [67], [68]]. For example mitochondrial DNA depletion in human cells, induced by expression of a dominant-negative mutant of mitochondrial DNA polymerase POLGdn, remodeled 1C metabolism especially serine biosynthesis and transsulfuration [64]. Similarly, in the Deletor mouse model of mitochondrial myopathy induced by mutation of the mitochondrial DNA helicase TWINKLE, depletion of mitochondrial DNA resulted in major changes in the folate cycle-dependent biosynthetic pathways, which was concomitant with an increase in the expression of MTHFD2 and MTHFD1L. Notably, in patients with PEO (progressive external ophthalmoplegia) due to TWINKLE mutations, cystathionine was increased in blood and muscle [65]. The expression profile of SKM in mice with compromised mitochondrial function due to UCP1 overexpression, revealed alterations in AA turnover, serine biosynthesis, polyamine and transsulfuration pathway and induction of MTHFD2 [67]. A similar metabolic response was found in OXPHOS-defective human muscles of MERRF patients with severe myopathy and in *Cox10*-ko mice, another mouse model of mitochondrial myopathy, including the utilization of AA for energy production, the induction of 1C metabolic genes, and the activation of the transsulfuration pathway [68].

We observed a lack of lipid droplet accumulation in muscles of mTKO mice even under HFD exposure as well as altered lipid metabolism included increased LPL protein and ACOT2, suggesting impaired beta-oxidation and energy storage. These findings align with hypermetabolism observed in mitochondrial diseases, where oxidative phosphorylation defects induce energy-consuming adaptations [69]. Such hypermetabolism likely contributes to the lean phenotype of our mTKO mice, and the protection from HFD-induced metabolic damage.

Hypermetabolism has been proposed as a consequence of OxPhos defects, and individuals with primary mitochondrial disorders are typically lean or underweight [69]. Patient cohorts show elevated resting EE, and patient derived fibroblasts display a cell autonomous doubling of energy use together with activation of the integrated stress response (ISR), proteostatic mechanisms, and redox detoxification pathways without evidence for generalized uncoupling. These observations are broadly consistent with current models suggesting that OxPhos defects may raise the energetic cost of living by engaging energy intensive adaptive pathways, even when ATP levels appear near normal [70].

In mTKO mice, the muscle may undergo metabolic remodeling toward energy intensive, stress responsive pathways, including MTHFD2 induction, one carbon rewiring, elevated NADPH, increased GSH/GSSG turnover, S-lactoylglutathione accumulation as a marker of enhanced glucose derived methylglyoxal detoxification, and cystathionine elevation, suggesting that glucose derived carbon might be diverted into 1C/transsulfuration based redox maintenance rather than ATP production. Concomitant alterations in acylcarnitines, ETFA/ACOT2, and reduced ATP levels could further reflect inefficient substrate oxidation and persistent redox repair demand.

Although indirect calorimetry quantifies EE via VO_2_ and VCO_2_, it may not adequately reflect ATP independent or oxygen minimal energetic costs, such as those arising from NADPH driven redox programs, proteostatic repair, and other hypermetabolism associated processes. We observed changes in cellular remodeling that may indicate that the lean phenotype of mTKO mice arises from muscle intrinsic, stress driven metabolic rewiring, paralleling mitochondrial stress responses described in established OxPhos defect models [64,65,67,68].

We used the Cre-LoxP system [71] to inactivate *Txnrd2* specifically in muscle fibers. While Cre is widely used as a genetic tool, it has been documented that Cre activity itself can exert deleterious effects, independent of the presence of loxP sites, including chromosomal instability, apoptosis, cardiac fibrosis, and behavioral changes. These effects have been reported for inducible, constitutive, and viral vector-mediated Cre expression alike [[72], [73], [74], [75]]. To address this concern experimentally, we evaluated whether the muscle phenotype in mTKO mice might in part be attributable to Cre expression rather than *Txnrd2* deficiency. For this purpose, we included both Cre bearing mice and rescue mice (mTKOTg), which express wild type *Txnrd2* in the mTKO background, and compared them with wild type and mTKO animals across multiple ages.

Our analyses reconfirmed that age progressive muscle atrophy is a consequence of *Txnrd2* loss, as muscle mass and gross architecture of Cre only and rescue mice remained intact relative to mTKO animals. Notably, however, Cre bearing and mTKOTg muscles displayed increased numbers of centrally nucleated fibers (CNFs) and occasional degenerating fibers. CNFs indicate preceding degeneration followed by regeneration. Yet CNFs have been shown to persist for years after injury and may therefore reflect a past regenerative event rather than ongoing degeneration [[76], [77], [78], [79]].

Additional mild regenerative features, such as focal infiltration, necrotic fibers, or small numbers of myoblasts, were observed in Cre bearing and rescue animals at slightly higher frequency than in wild type controls. These findings suggest that small, recurrent muscle injuries that undergo repair may contribute to the accumulation of CNFs, and that nuclear repositioning may remain incomplete in these contexts.

Importantly, the ACTA1/HSA promoter used in our model drives Cre expression in mature myofibers but shows little to no activity in satellite cells, the muscle stem cell population [34]. Therefore, the regenerative capacity of satellite cells, and thus the ability to repair mild or chronic injury, should remain unaffected by Cre expression or by *Txnrd2* deletion in myofibers.

Interestingly, mTKO mice maintain a comparatively mild clinical phenotype despite progressive atrophy: they remain mobile, feed and drink independently, and at least the males retain near normal lifespan. This raises the possibility that preserved satellite cell function contributes to the phenotype, as described in other models (e.g., Smn muscle specific knockout), where intact satellite cells mitigated disease severity [80]. Nevertheless, the influence of satellite cells on muscle disease phenotypes is complex and context dependent and has not always been shown to be beneficial [81,82].

Taken together, we acknowledge that Cre associated effects exist and may contribute subtle regenerative signatures, but our comparative data across multiple genotypes and ages strongly indicate that the dominant driver of muscle atrophy in our model is *Txnrd2* deficiency, while Cre related changes represent a separate, mild regenerative background that does not account for the profound atrophy phenotype in mTKO mice.

## 5 Conclusion

In conclusion, *Txnrd2* deletion in skeletal muscle disrupts redox balance and energy metabolism, yielding a lean yet frail phenotype characterized by muscle fatigue. mTKO mice displayed mitochondrial dysfunction and biochemical perturbations in lipid metabolism as well as redox control, which highlights the plurality of pathways involved in muscle homeostasis that may inform therapeutic strategies for redox-related diseases.

## Funding sources

This study was supported in part by a grant from the German Federal Ministry of Education and Research (BMBF) to the German Center for Diabetes Research (DZD e.V.).

## CRediT authorship contribution statement

**Claudia Kiermayer:** Conceptualization, Data curation, Formal analysis, Investigation, Project administration, Validation, Visualization, Writing – original draft. **Rebecca Erdelen:** Data curation, Formal analysis, Investigation, Validation, Writing – review & editing. **Sonja C. Schriever:** Data curation, Formal analysis, Investigation, Validation, Visualization, Writing – review & editing. **Ramona Böhm:** Formal analysis, Investigation, Validation, Writing – review & editing. **Cornelia Prehn:** Data curation, Formal analysis, Investigation, Validation, Writing – review & editing. **Anna Artati:** Data curation, Formal analysis, Investigation, Validation, Writing – review & editing. **Maximilian Kleinert:** Data curation, Formal analysis, Investigation, Validation, Writing – review & editing. **Manuel Miller:** Formal analysis, Visualization, Writing – review & editing. **Kenneth A. Dyar:** Project administration, Resources, Writing – review & editing. **Roland M. Schmid:** Formal analysis, Methodology, Resources, Validation, Writing – review & editing. **Paul T. Pfluger:** Formal analysis, Methodology, Resources, Validation, Writing – review & editing. **Jerzy Adamski:** Formal analysis, Methodology, Resources, Validation, Writing – original draft, Writing – review & editing. **Markus Brielmeier:** Conceptualization, Resources, Supervision, Validation, Writing – review & editing.

## Declaration of competing interest

The authors declare that they have no known competing financial interests or personal relationships that could have appeared to influence the work reported in this paper.

## Data Availability

Source data are provided with this paper.
