# Supplementary material for: Txnrd2 loss in skeletal muscle causes muscle atrophy and drives leanness and obesity resistance
Source: Redox Biol. 2026 Apr 8;93:104165. doi: 10.1016/j.redox.2026.104165 (PMC13099521; doi:10.1016/j.redox.2026.104165)
Supplement: Multimedia component 1 [file mmc1.docx]

### Supplementary Information: *Txnrd2* loss in skeletal muscle causes atrophy and drives leanness and obesity resistance.

### Contains: Supplementary Figures S1–S7.

**Authors:**

Claudia Kiermayer ^a,*^, Rebecca Erdelen ^a^, Sonja C. Schriever ^b,c^, Ramona Böhm ^a,1^, Cornelia Prehn ^d^, Anna Artati ^d^, Maximilian Kleinert ^c,e,f^, Manuel Miller ^a^, Kenneth A. Dyar ^c,g^, Roland M. Schmid ^h^, Paul T. Pfluger ^b,c,i^, Jerzy Adamski ^j,k,l^, Markus Brielmeier ^a^

**Author information:**

^a^ Core Facility Laboratory Animal Services (CF-LAS), Helmholtz Munich, German Research Center for Environmental Health, Neuherberg, Germany.

^b^ Research Unit Neurobiology of Diabetes, Institute for Diabetes and Obesity, Helmholtz Munich, German Research Center for Environmental Health, Neuherberg, Germany.

^c^ German Center for Diabetes Research (DZD), Neuherberg, Germany.

^d^ Metabolomics and Proteomics Core (CF-MPC), Helmholtz Munich, German Research Center for Environmental Health, Neuherberg, Germany.

^e^ Department of Molecular Physiology of Exercise and Nutrition, German Institute of Human Nutrition (DifE) Potsdam-Rehbruecke, Nuthetal, Germany.

^f^ Institute of Nutritional Sciences, University of Potsdam, Nuthetal, Germany.

^g^ Metabolic Physiology, Institute for Diabetes and Cancer, Helmholtz Munich, German Research Center for Environmental Health, Neuherberg, Germany.

^h^ Department of Internal Medicine II, TUM School of Medicine and Health, TUM

University Hospital, Technical University of Munich, Munich, Germany.

^i^ Division of Neurobiology of Diabetes, TUM School of Medicine & Health, Technical University of Munich, Munich, Germany.

^j^ Institute of Experimental Genetics, Helmholtz Munich, German Research Center for Environmental Health, Neuherberg, Germany.

^k^ Department of Biochemistry, Yong Loo Lin School of Medicine, National University of Singapore, Singapore.

^l^ Institute of Biochemistry, Faculty of Medicine, University of Ljubljana, Ljubljana, Slovenia

^1^ Present address: University of Hohenheim, Stuttgart, Germany.

^*^Corresponding author. E-Mail address: [claudia.kiermayer@helmholtz-munich.de](mailto:claudia.kiermayer@helmholtz-munich.de)


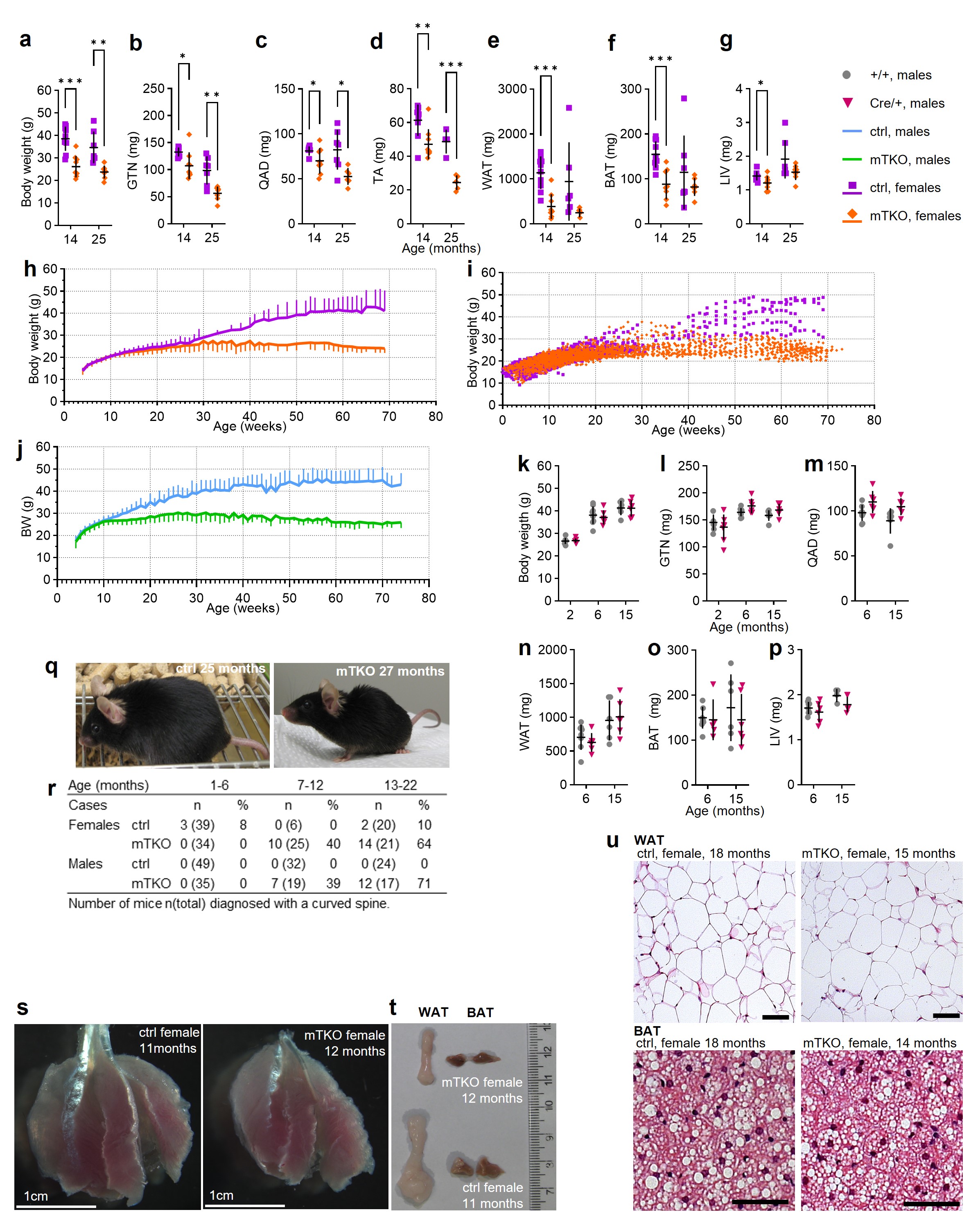


**Supplementary Figure S1.**

**Supplementary Figure S1. The lean phenotype induced by *Txnrd2*-deficiency is absent in Cre controls.**

**a**-**g**, Body and tissue weights of female mice at different ages as indicated. Asterisks * indicate statistical significance of mTKO (fl/fl; Cre) versus ctrl (fl/fl) of the same age. **h**-**j**, Body weight growth curves of (**h**, **i**) female mice (mean ± SD (**h**) and body weights of individual mice (**i**)) and (**j**) male mice (same data as in Fig.1c). **k**-**p**, Body and tissue weights of male mice at different ages as indicated **q**, Representative images of ko versus ctrl males, highlighting the curvature of the spine. **r**, Number of male and female mice diagnosed with a curved spine for the indicated ages. **s** and **t**, Images of (**s**) GTN and (**t**) WAT and BAT isolated from ctrl and mTKO female mice. **u**, H&E-stained paraffin sections of WAT and BAT. Age as indicated. **a**-**g** and **k**-**p**, Results are mean ± SD. *P* values by unpaired two-tailed *t*-test. Statistical significance was defined as *P* ≤ 0.05 (*), *P* ≤ 0.01 (**) or *P* ≤ 0.001 (***). Genotypes as indicated.


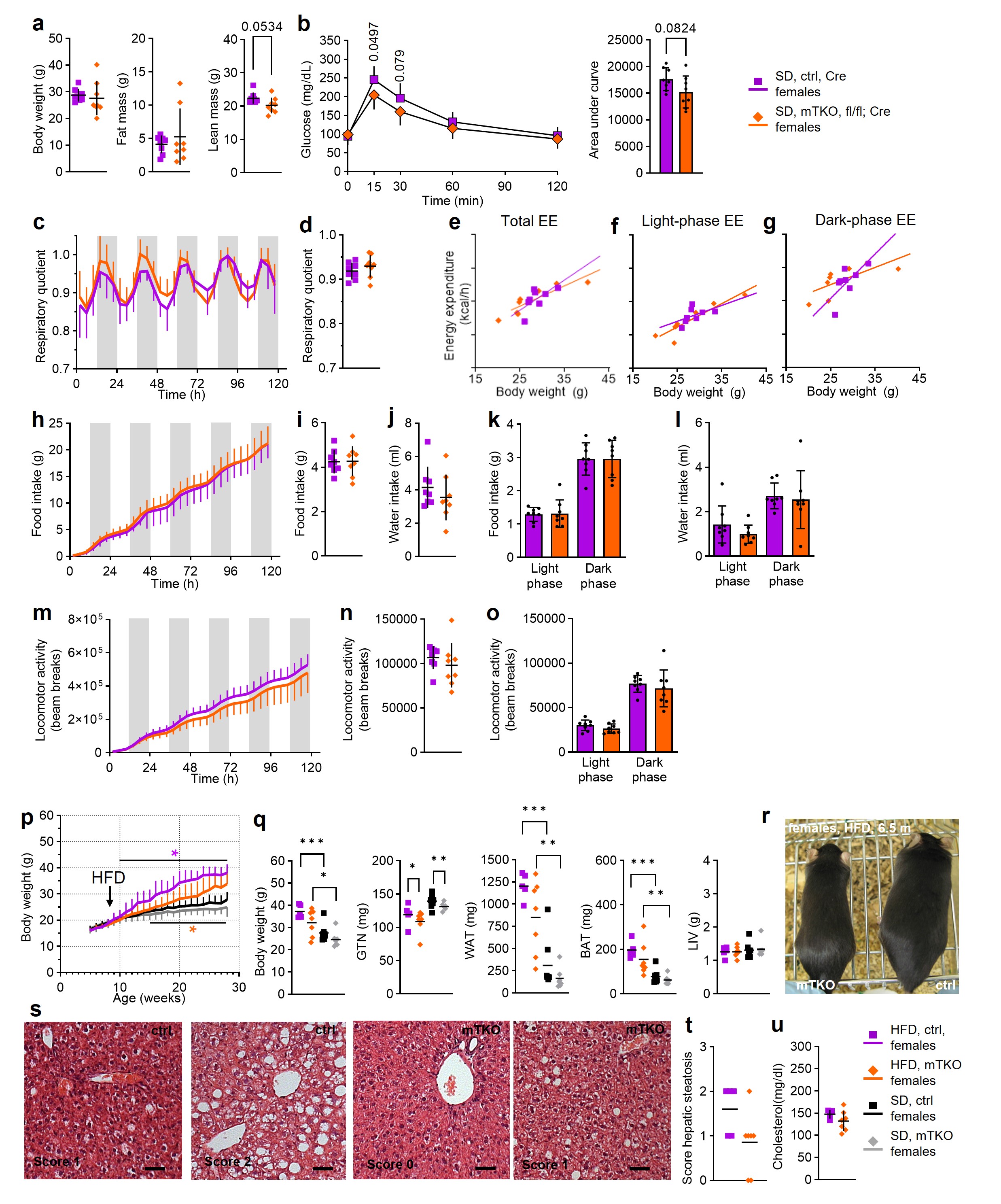


**Supplementary Figure S2.**

**Supplementary Figure S2. mTKO female mice are protected from diet-induced obesity.**

**a**, Body weight, fat and lean mass of mTKO versus ctrl. **b**, Glucose tolerance test (GTT). **c**, Respiratory exchange ratio (RER) over 5 days, **d**, relative daily RER **e**, total energy expenditure (EE) **f**, light-phase EE **g**, dark-phase EE, **h,** cumulative food intake, **i,** average daily food intake, **j,** average daily water intake **k**, day and night food intake, **l**, day and night water intake, **m**, cumulative locomotor activity, **n**, average daily locomotor activity and **o**, average day and night locomotor activity were assessed in female mTKO mice compared to ctrl in indirect calorimetry chambers. **a**-**o**, n=8 female mice for both genotypes. **p**, Body weight growth curves of female mice receiving a high-fat diet (HFD) from the age of 8 weeks on, compared to females on standard chow diet (SD) (n=5-8 for ctrl, n=5-7 for mTKO on HFD and n=8-16 for ctrl, n=7-15 for mTKO on SD). **q**, Body weight and isolated tissue weights of female mice after HFD or SD feeding (n=5 for control, n=7 for mTKO on HFD and n=8 for control, n=7 for mTKO on SD). **r**, Representative images of female mice on HFD. **s**, Representative images of H&E-stained liver paraffin-sections from HFD-fed female mice. Scale bar, 50 µm. **t**, Scoring of hepatic steatosis based on H&E-staining of livers form HFD-fed female mice (n=5 sections form 5 animals for control, n=7 sections form 7 animals for mTKO). **u**, Blood cholesterol levels after HFD feeding (n=4 animals for control, n=7 animals for mTKO). Genotypes as indicated.


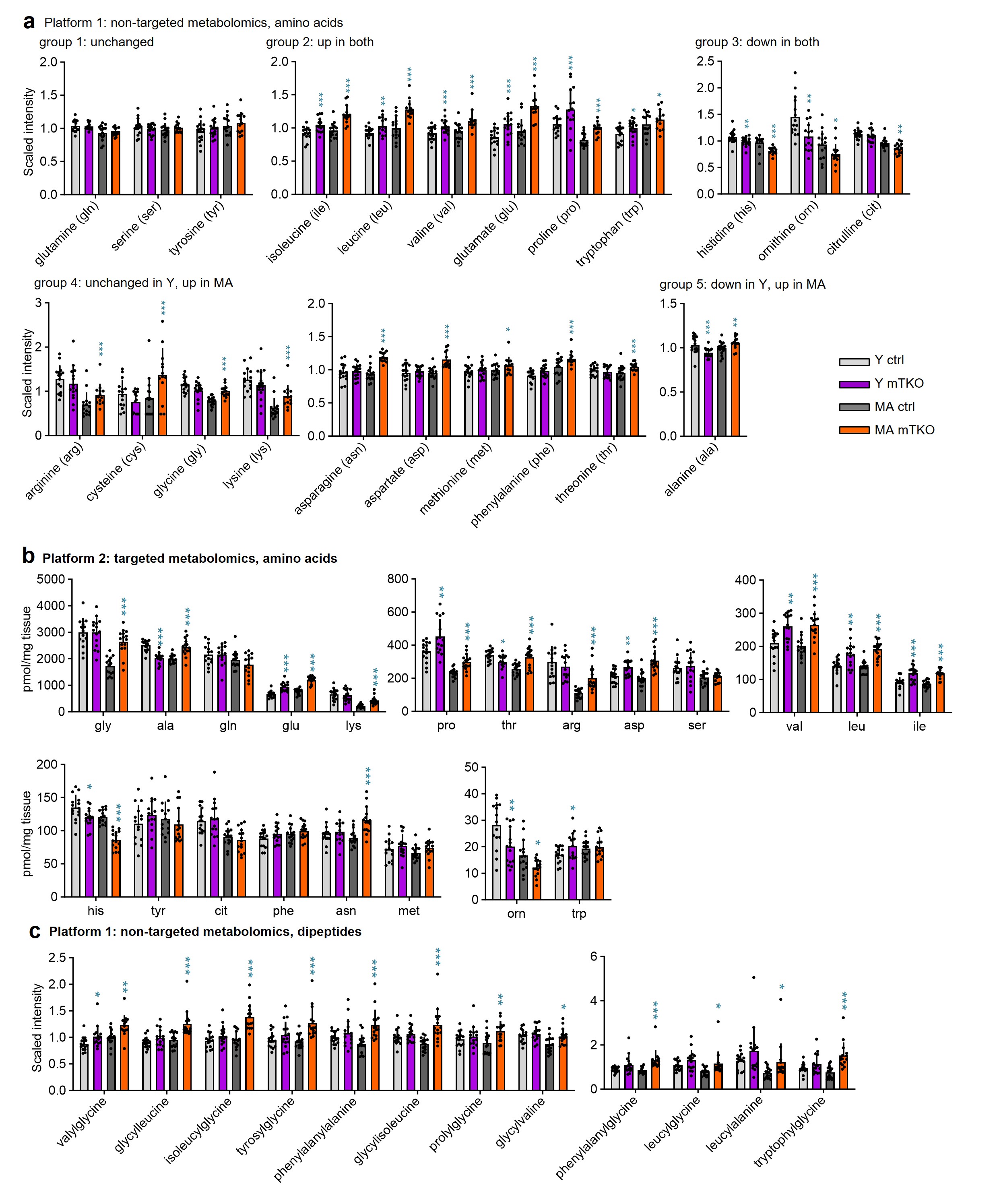


**Supplementary Figure S3.**

**Supplementary Figure S3. Metabolite alterations are highly reproducible.**

**a**, and **b**, Abundance of amino acids (AA) as evaluated by (**a**) non-targeted and (**b**) targeted metabolomics. AAs were either (**a**) assigned to different categories according to direction of changes (non-targeted) or (**b**) sorted by concentration (targeted). **c**, Abundance of dipeptides as evaluated by non-targeted metabolomics. Statistical significance was evaluated using Student's unpaired two-tailed T-test. Asterisks * indicate statistical significance of mTKO versus ctrl of the same age. Y, young; MA, middle-aged; Statistical significance was defined as *P* ≤ 0.05 (*), *P* ≤ 0.01 (**) or *P* ≤ 0.001 (***). Error bars represent SD. Different y-axes were used to improve visual clarity.


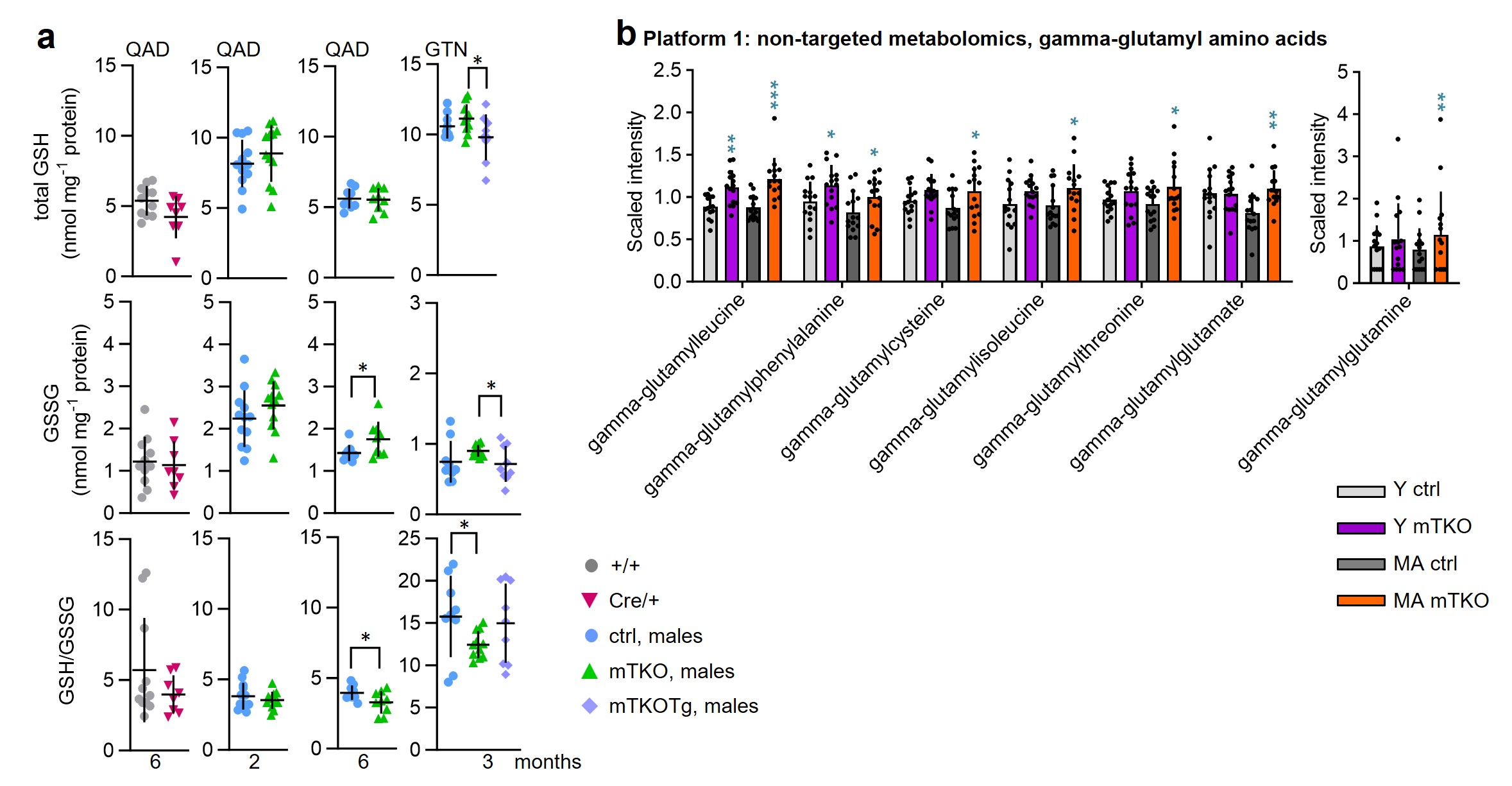


**Supplementary Figure S4. Alterations in GSH metabolism in mTKO muscle.**

**a**, GSH and GSSG amounts measured in GTN and QAD tissue as indicated, and GSH to GSSG ratio calculated from this. Age and genotype as indicated **b**, Abundance of gamma-glutamyl AAs as evaluated by non-targeted metabolomics. Asterisks * indicate statistical significance of mTKO versus ctrl of the same age.


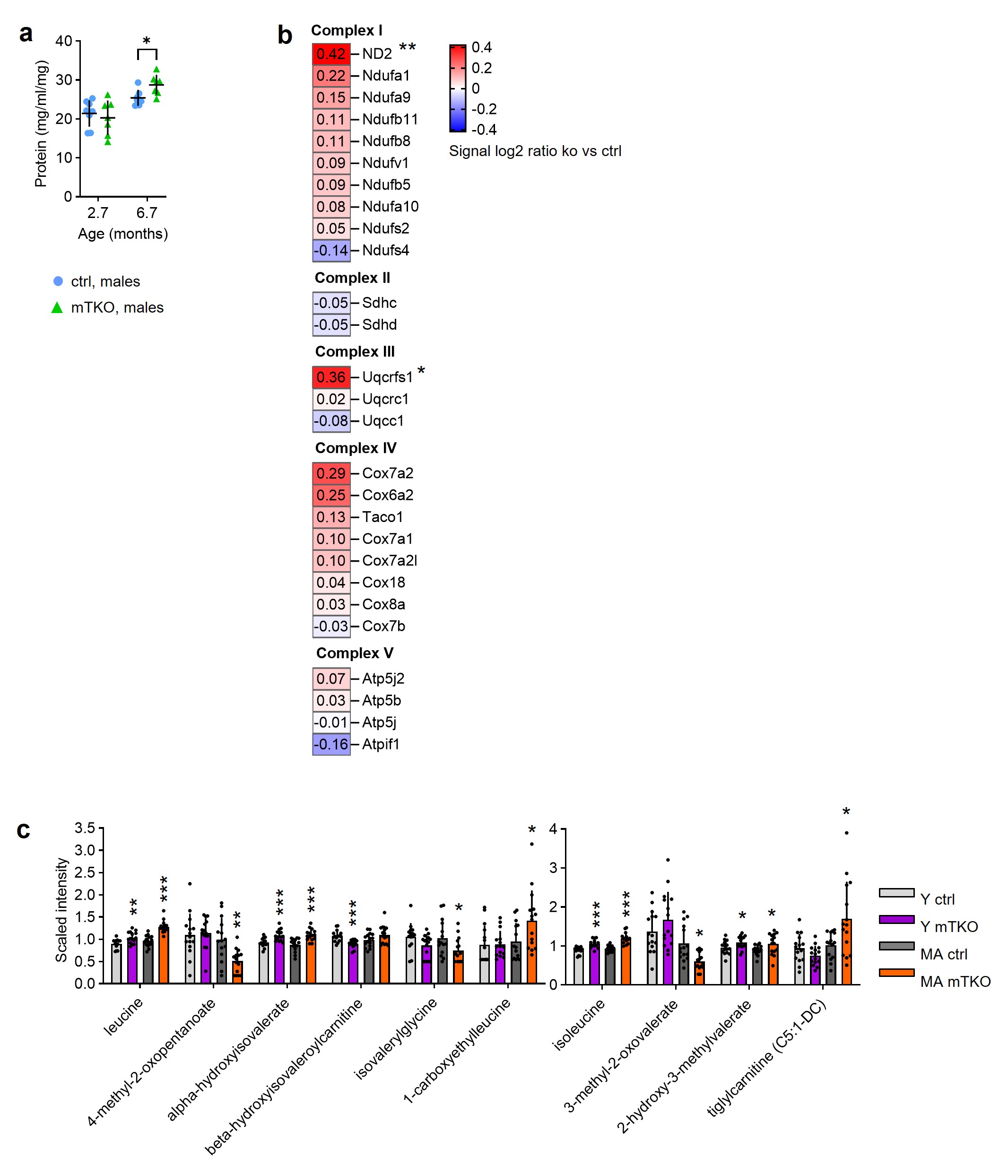


**Supplementary Figure S5.**

**Supplementary Figure S5. *Txnrd2*-deficiency induces mitochondrial dysfunction in skeletal muscle.**

**a,** Protein concentration of mitochondrial fraction normalized to input muscle mass. 2.7 months: n=8 for ctrl and 7 for mTKO; 6.7 months: n=7 for ctrl and mTKO. 6.7 months **b**, Heatmap showing gene expression changes of genes encoding components of the respiratory chain. Transcript levels analysed in GTN from mTKO mice versus ctrl. Genes are presented in decreasing order of signal log2 ratio. Transcript levels are displayed in red when upregulated or in blue when downregulated. n=5 for ctrl and mTKO. **c**, Abundance of leucine and isoleucine and respective degradation products as evaluated by non-targeted metabolomics. The asterisks * indicate statistical significance of mTKO versus ctrl of the same age. Statistical significance was defined as *P* ≤ 0.05 (*), *P* ≤ 0.01 (**) or *P* ≤ 0.001 (***).


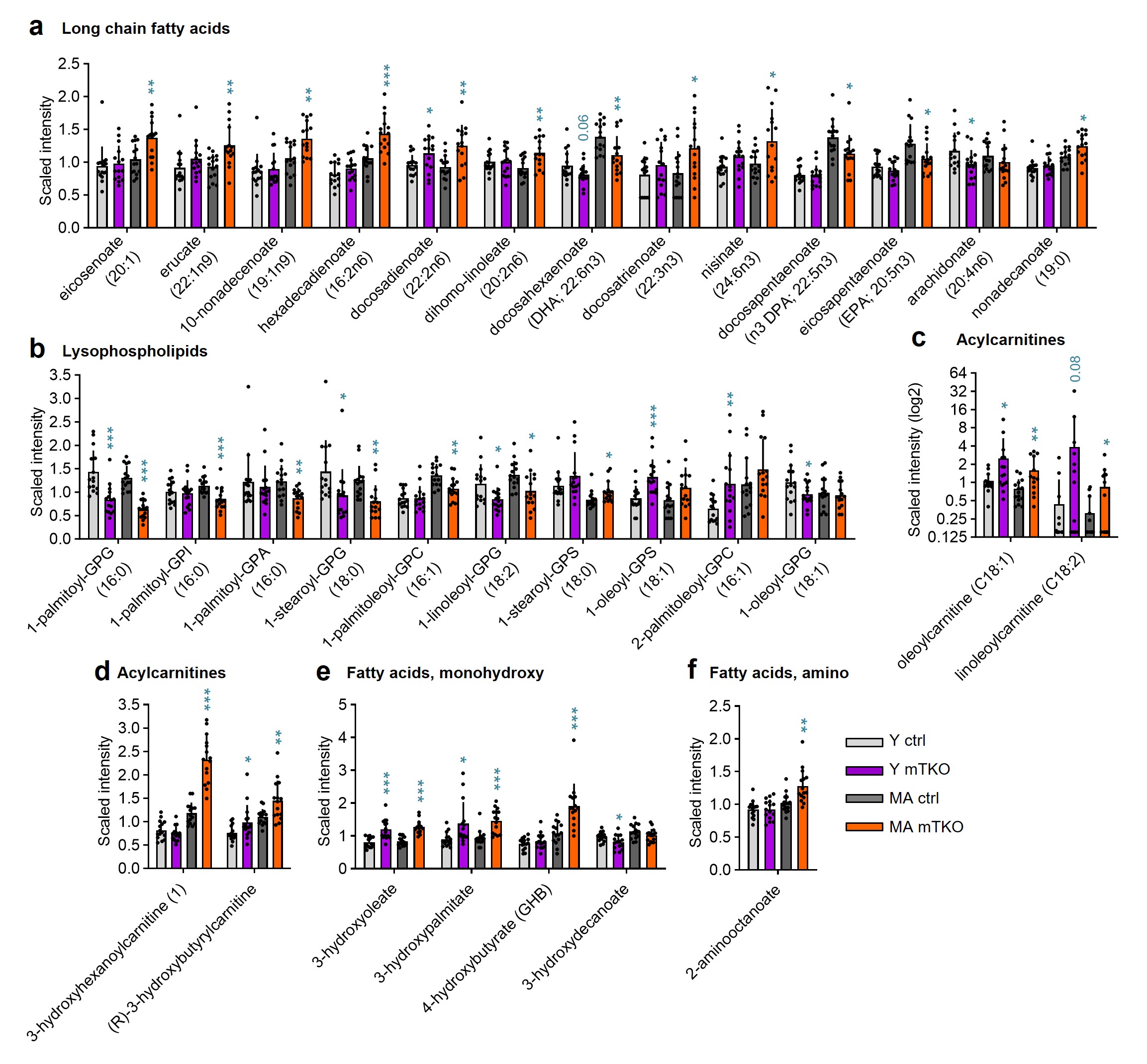


**Supplementary Figure S6. *Txnrd2*-deficiency alters amount of several lipid species in muscle tissue.**

**a**-**f,** Abundance of lipids as evaluated by non-targeted metabolomics. (**a**) Long chain fatty acids, (**b**) lysopholpholipids, (**c**,**d**) acylcarnitines, (**e,f**) fatty acids. The asterisks * indicate statistical significance of mTKO versus ctrl of the same age. Y, young; MA, middle-aged; Statistical significance was defined as *P* ≤ 0.05 (*), *P* ≤ 0.01 (**) or *P* ≤ 0.001 (***). To enhance visual clarity, different y-axes were used.


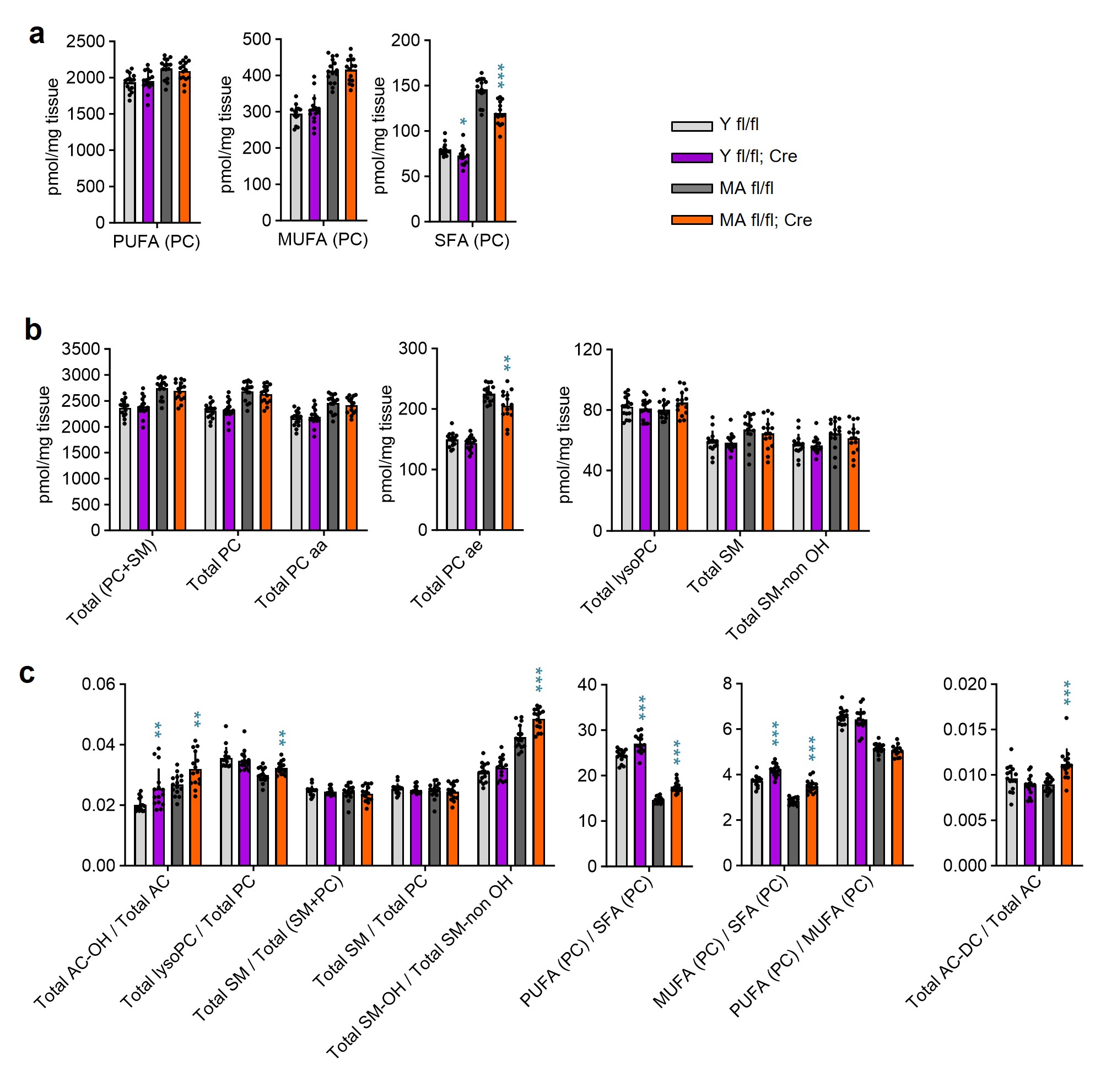


**Supplementary Figure S7.**

**Supplementary Figure S7. Altered lipid fractions in GTN from mTKO mice.**

**a**-**c**, Total amount of certain lipid fractions and their ratio to each other, as evaluated by targeted metabolomics. (**a**) Sum of poly-unsaturated glycerophosphocholines (PUFA), mono-unsaturated glycerophosphocholines (MUFA), and saturated glycerophosphocholines (SFA). (**b**), Sum of choline containing phospholipids (total (PC+SM)), glycerophosphocholines (total PC), diacyl-glycerophosphocholines (total PC aa), glycerophosphocholines plasmalogens (total PCae), lysoglycerophosphocholines (total lysoPC), ceramide phosphocholines (sphingomyelins, total SM) and non-hydroxylated ceramide phosphochlines (total SM-non OH). (**c**), Ratio of different lipid fractions to each other. AC, acylcarnitines; AC-DC, dicarboxyacylcarnitines; AC-OH, hydroxylated acylcarnitines; The asterisks * indicate statistical significance of mTKO versus ctrl of the same age. Y, young; MA, middle-aged; Statistical significance was defined as *P* ≤ 0.05 (*), *P* ≤ 0.01 (**) or *P* ≤ 0.001 (***). Note that to enhance visual clarity, different y-axes were used.
