# Supplementary material for: Txnrd2 loss in skeletal muscle causes muscle atrophy and drives leanness and obesity resistance
Source: Redox Biol. 2026 Apr 8;93:104165. doi: 10.1016/j.redox.2026.104165 (PMC13099521; doi:10.1016/j.redox.2026.104165)
Supplement: Multimedia component 2 [file mmc2.docx]

**Description of Supplementary Tables**

**Supplementary Table 1.**
*Affymetrix-based transcriptomic profiling of skeletal muscle from ctrl and mTKO mice.*
Gene expression data from Affymetrix microarrays comparing gastrocnemius muscle of ctrl and mTKO males (n=5 biological replicates per group; 1-5: ctrl, 6-10: mTKO). Includes log2-transformed signal intensities, average expression, fold changes (mTKO vs. ctrl), and p-values. Annotations cover gene IDs, symbols, descriptions, chromosomal locations, and transcript details, along with GO terms, pathways, and protein domains.

**Supplementary Table 2.**
*Differential metabolite abundance in ctrl versus mTKO young mice.*

Metabolites identified through non-targeted metabolomics in gastrocnemius muscle from 2-month-old male mice (young, Y), comparing control (ctrl) and muscle-specific *Txnrd2* knockout (mTKO) groups. For each metabolite, the biochemical name, super pathway, and sub pathway are provided, along with the mean abundance in each group, the p-value, and the fold change (mTKO/ctrl). Individual replicate values (n = 15 per group) are included. Fold changes <1 indicate decreased abundance in mTKO, while values >1 indicate increased abundance. P-values were calculated using unpaired two-tailed t-tests

**Supplementary Table 3.**
*Differential metabolite abundance in ctrl versus mTKO middle-aged mice.*

Metabolites identified through non-targeted metabolomics in gastrocnemius muscle from 6-month-old male mice (middle-aged; MA), comparing control (ctrl) and muscle-specific *Txnrd2* knockout (mTKO) groups. For each metabolite, the biochemical name, super pathway, and sub pathway are provided, along with the mean abundance in each group, the p-value from statistical comparison, and the fold change (mTKO/Ctrl). Individual replicate values (n = 15 per group) are included. Fold changes <1 indicate decreased abundance in mTKO, while values >1 indicate increased abundance. P-values were calculated using unpaired two-tailed t-tests.

**Supplementary Table 4.**
*Differential metabolite abundance ctrl versus mTKO young mice.*Metabolites quantified by targeted metabolomics in gastrocnemius muscle tissue from young control (ctrl) and muscle-specific *Txnrd2* knockout (mTKO) mice. For each metabolite, the biochemical name is provided along with the mean concentration in each group (expressed in pmol/mg tissue), the p-value, and the fold change (mTKO/ctrl). Individual replicate values (n = 15 per group) are included. Fold changes <1 indicate decreased abundance in mTKO, while values >1 indicate increased abundance. P-values were calculated using unpaired two-tailed t-tests.

**Supplementary Table 5.**
*Differential metabolite abundance in ctrl versus mTKO middle-aged mice.*Metabolites quantified through targeted metabolomics of gastrocnemius muscle tissue from middle-aged control (ctrl) and muscle-specific *Txnrd2* knockout (mTKO) mice. For each metabolite, the biochemical name is provided along with the mean concentration in each group (expressed in pmol/mg tissue), the p-value, and the fold change (mTKO/Ctrl). Individual replicate values (n = 15 per group) are included. Fold changes <1 indicate decreased abundance in mTKO, while values >1 indicate increased abundance. P-values were

calculated using unpaired two-tailed t-tests.

**Replicate Assignment for Supplementary Table 2-5.**

| Replicate ID | Group | Age Category |
| --- | --- | --- |
| 1–15 | ctrl | young |
| 16–30 | mTKO | young |
| 31–45 | ctrl | middle-aged |
| 46–60 | mTKO | middle-aged |

**Metabolite Abbreviations and Full Names (Biocrates AbsoluteIDQ® p180 Kit) for Supplementary Table 4 and 5.**

**Amino Acids**

| Abbreviation | Full Name |
| --- | --- |
| Ala | Alanine |
| Arg | Arginine |
| Asn | Asparagine |
| Asp | Aspartate |
| Cit | Citrulline |
| Gln | Glutamine |
| Glu | Glutamate |
| Gly | Glycine |
| His | Histidine |
| Ile | Isoleucine |
| Leu | Leucine |
| Lys | Lysine |
| Met | Methionine |
| Orn | Ornithine |
| Phe | Phenylalanine |
| Pro | Proline |
| Ser | Serine |
| Thr | Threonine |
| Trp | Tryptophan |
| Tyr | Tyrosine |
| Val | Valine |

**Biogenic Amines**

| Abbreviation | Full Name |
| --- | --- |
| Ac-Orn | Acetylornithine |
| ADMA | Asymmetric dimethylarginine |
| alpha-AAA | Alpha-aminoadipic acid |
| Carnosine | Carnosine |
| Creatinine | Creatinine |
| Histamine | Histamine |
| Met-SO | Methionine sulfoxide |
| Putrescine | Putrescine |
| Serotonin | Serotonin |
| Spermidine | Spermidine |
| Spermine | Spermine |
| t4-OH-Pro | trans-4-Hydroxyproline |
| Taurine | Taurine |

**Acylcarnitines**

| Abbreviation | Full Name |
| --- | --- |
| C0 | Free Carnitine |
| C2 | Acetylcarnitine |
| C3 | Propionylcarnitine |
| C4 | Butyrylcarnitine |
| C5 | Valerylcarnitine |
| C5-OH (C3-DC-M) | Hydroxyvalerylcarnitine (Methylmalonylcarnitine) |
| C5-DC (C6-OH) | Glutarylcarnitine (Hydroxyhexanoylcarnitine) |
| C3-DC (C4-OH) | Hydroxybutyrylcarnitine |
| C14 | Tetradecanoylcarnitine |
| C14:1 | Tetradecenoylcarnitine |
| C14:1-OH | Hydroxytetradecenoylcarnitine |
| C16 | Hexadecanoylcarnitine |
| C16-OH | Hydroxyhexadecanoylcarnitine |
| C16:1-OH | Hydroxyhexadecenoylcarnitine |
| C16:2 | Hexadecadienoylcarnitine |
| C18 | Octadecanoylcarnitine |
| C18:1 | Octadecenoylcarnitine |
| C18:1-OH | Hydroxyoctadecenoylcarnitine |
| C18:2 | Octadecadienoylcarnitine |

**Glycerophospholipids**

| Abbreviation | Full Name |
| --- | --- |
| lysoPC a C16:0 | lysoPhosphatidylcholine acyl C16:0 |
| lysoPC a C16:1 | lysoPhosphatidylcholine acyl C16:1 |
| lysoPC a C17:0 | lysoPhosphatidylcholine acyl C17:0 |
| lysoPC a C18:0 | lysoPhosphatidylcholine acyl C18:0 |
| lysoPC a C18:1 | lysoPhosphatidylcholine acyl C18:1 |
| lysoPC a C18:2 | lysoPhosphatidylcholine acyl C18:2 |
| lysoPC a C20:3 | lysoPhosphatidylcholine acyl C20:3 |
| lysoPC a C20:4 | lysoPhosphatidylcholine acyl C20:4 |
| lysoPC a C26:0 | lysoPhosphatidylcholine acyl C26:0 |
| lysoPC a C26:1 | lysoPhosphatidylcholine acyl C26:1 |
| lysoPC a C28:0 | lysoPhosphatidylcholine acyl C28:0 |
| lysoPC a C28:1 | lysoPhosphatidylcholine acyl C28:1 |
| PC aa C24:0 | Phosphatidylcholine diacyl C24:0 |
| PC aa C28:1 | Phosphatidylcholine diacyl C28:1 |
| PC aa C30:0 | Phosphatidylcholine diacyl C30:0 |
| PC aa C30:2 | Phosphatidylcholine diacyl C30:2 |
| PC aa C32:0 | Phosphatidylcholine diacyl C32:0 |
| PC aa C32:1 | Phosphatidylcholine diacyl C32:1 |
| PC aa C32:2 | Phosphatidylcholine diacyl C32:2 |
| PC aa C32:3 | Phosphatidylcholine diacyl C32:3 |
| PC aa C34:1 | Phosphatidylcholine diacyl C34:1 |
| PC aa C34:2 | Phosphatidylcholine diacyl C34:2 |
| PC aa C34:3 | Phosphatidylcholine diacyl C34:3 |
| PC aa C34:4 | Phosphatidylcholine diacyl C34:4 |
| PC aa C36:0 | Phosphatidylcholine diacyl C36:0 |
| PC aa C36:1 | Phosphatidylcholine diacyl C36:1 |
| PC aa C36:2 | Phosphatidylcholine diacyl C36:2 |
| PC aa C36:3 | Phosphatidylcholine diacyl C36:3 |
| PC aa C36:4 | Phosphatidylcholine diacyl C36:4 |
| PC aa C36:5 | Phosphatidylcholine diacyl C36:5 |
| PC aa C36:6 | Phosphatidylcholine diacyl C36:6 |
| PC aa C38:0 | Phosphatidylcholine diacyl C38:0 |
| PC aa C38:1 | Phosphatidylcholine diacyl C38:1 |
| PC aa C38:3 | Phosphatidylcholine diacyl C38:3 |
| PC aa C38:4 | Phosphatidylcholine diacyl C38:4 |
| PC aa C38:5 | Phosphatidylcholine diacyl C38:5 |
| PC aa C38:6 | Phosphatidylcholine diacyl C38:6 |
| PC aa C40:2 | Phosphatidylcholine diacyl C40:2 |
| PC aa C40:3 | Phosphatidylcholine diacyl C40:3 |
| PC aa C40:4 | Phosphatidylcholine diacyl C40:4 |
| PC aa C40:5 | Phosphatidylcholine diacyl C40:5 |
| PC aa C40:6 | Phosphatidylcholine diacyl C40:6 |
| PC aa C42:1 | Phosphatidylcholine diacyl C42:1 |
| PC aa C42:2 | Phosphatidylcholine diacyl C42:2 |
| PC aa C42:4 | Phosphatidylcholine diacyl C42:4 |
| PC aa C42:5 | Phosphatidylcholine diacyl C42:5 |
| PC aa C42:6 | Phosphatidylcholine diacyl C42:6 |
| PC ae C30:0 | Phosphatidylcholine acyl-alkyl C30:0 |
| PC ae C30:1 | Phosphatidylcholine acyl-alkyl C30:1 |
| PC ae C32:1 | Phosphatidylcholine acyl-alkyl C32:1 |
| PC ae C32:2 | Phosphatidylcholine acyl-alkyl C32:2 |
| PC ae C34:0 | Phosphatidylcholine acyl-alkyl C34:0 |
| PC ae C34:1 | Phosphatidylcholine acyl-alkyl C34:1 |
| PC ae C34:2 | Phosphatidylcholine acyl-alkyl C34:2 |
| PC ae C34:3 | Phosphatidylcholine acyl-alkyl C34:3 |
| PC ae C36:0 | Phosphatidylcholine acyl-alkyl C36:0 |
| PC ae C36:1 | Phosphatidylcholine acyl-alkyl C36:1 |
| PC ae C36:2 | Phosphatidylcholine acyl-alkyl C36:2 |
| PC ae C36:3 | Phosphatidylcholine acyl-alkyl C36:3 |
| PC ae C36:4 | Phosphatidylcholine acyl-alkyl C36:4 |
| PC ae C36:5 | Phosphatidylcholine acyl-alkyl C36:5 |
| PC ae C38:0 | Phosphatidylcholine acyl-alkyl C38:0 |
| PC ae C38:1 | Phosphatidylcholine acyl-alkyl C38:1 |
| PC ae C38:2 | Phosphatidylcholine acyl-alkyl C38:2 |
| PC ae C38:3 | Phosphatidylcholine acyl-alkyl C38:3 |
| PC ae C38:4 | Phosphatidylcholine acyl-alkyl C38:4 |
| PC ae C38:5 | Phosphatidylcholine acyl-alkyl C38:5 |
| PC ae C38:6 | Phosphatidylcholine acyl-alkyl C38:6 |
| PC ae C40:1 | Phosphatidylcholine acyl-alkyl C40:1 |
| PC ae C40:2 | Phosphatidylcholine acyl-alkyl C40:2 |
| PC ae C40:3 | Phosphatidylcholine acyl-alkyl C40:3 |
| PC ae C40:4 | Phosphatidylcholine acyl-alkyl C40:4 |
| PC ae C40:5 | Phosphatidylcholine acyl-alkyl C40:5 |
| PC ae C40:6 | Phosphatidylcholine acyl-alkyl C40:6 |
| PC ae C42:1 | Phosphatidylcholine acyl-alkyl C42:1 |
| PC ae C42:2 | Phosphatidylcholine acyl-alkyl C42:2 |
| PC ae C42:3 | Phosphatidylcholine acyl-alkyl C42:3 |
| PC ae C42:4 | Phosphatidylcholine acyl-alkyl C42:4 |
| PC ae C44:3 | Phosphatidylcholine acyl-alkyl C44:3 |
| PC ae C44:4 | Phosphatidylcholine acyl-alkyl C44:4 |
| PC ae C44:5 | Phosphatidylcholine acyl-alkyl C44:5 |
| PC ae C44:6 | Phosphatidylcholine acyl-alkyl C44:6 |

**Sphingomyelins**

| Abbreviation | Full Name |
| --- | --- |
| SM (OH) C22:1 | Hydroxysphingomyelin C22:1 |
| SM (OH) C22:2 | Hydroxysphingomyelin C22:2 |
| SM (OH) C24:1 | Hydroxysphingomyelin C24:1 |
| SM C16:0 | Sphingomyelin C16:0 |
| SM C16:1 | Sphingomyelin C16:1 |
| SM C18:0 | Sphingomyelin C18:0 |
| SM C18:1 | Sphingomyelin C18:1 |
| SM C24:0 | Sphingomyelin C24:0 |
| SM C24:1 | Sphingomyelin C24:1 |

**Hexoses**

| Abbreviation | Full Name |
| --- | --- |
| H1 | Hexoses (sum of hexoses including glucose) |

**Supplementary Table 6.**
Source data for Figures 1–9 of the main manuscript and for Supplementary Figures S1–S4.
